# Using Φ_PSII_ and leaf temperature as indicators of non-steady-state photosynthesis and stomatal conductance during stepwise changes in light intensity

**DOI:** 10.1093/jxb/erag231

**Published:** 2026-05-16

**Authors:** Elahe Javadi Asayesh, Leo F M Marcelis, Liana G Acevedo-Siaca, Elias Kaiser

**Affiliations:** Horticulture and Product Physiology, Department of Plant Sciences, Wageningen University & Research, PB 6708, Wageningen, The Netherlands; Horticulture and Product Physiology, Department of Plant Sciences, Wageningen University & Research, PB 6708, Wageningen, The Netherlands; Horticulture and Product Physiology, Department of Plant Sciences, Wageningen University & Research, PB 6708, Wageningen, The Netherlands; Horticulture and Product Physiology, Department of Plant Sciences, Wageningen University & Research, PB 6708, Wageningen, The Netherlands; Research Institute of Agriculture and Life Sciences, Seoul National University, Seoul, Republic of Korea; University of Cambridge, UK

**Keywords:** Chlorophyll fluorescence, CO_2_ assimilation, leaf temperature, non-steady-state photosynthesis, photosynthetic induction, stomatal conductance

## Abstract

Quantifying the kinetics of net CO_2_ assimilation (*A*) and stomatal conductance (*g*_s_) under fluctuating light typically relies on gas exchange measurements, which are slow and thus unsuited for high-throughput phenotyping. As a result, faster, non-invasive phenotyping methods are needed to further evaluate these traits at a larger scale. However, first the relationship between non-steady-state parameters must be examined in greater detail. In this study, we aimed to determine whether variations in non-steady-state values of chlorophyll fluorescence and leaf temperature reflect differences in key gas exchange traits under fluctuating light conditions. Here, the correlations between the times required for a change in non-steady-state *A*, *g_s_*, operating efficiency of PSII (Φ_PSII_), and leaf temperature (*T*_leaf_) during stepwise changes in light intensity were evaluated across nine plant species. Both steady-state and non-steady-state photosynthetic traits varied significantly among species. Overall, we found significant positive correlations between non-steady-state *A* and Φ_PSII_ for time to 50% and 90% of final steady-state values (*t*_50_; *r*^2^ = 0.70) and (*t*_90_; *r*^2^ = 0.33). The *t*_90_ of *g*_s_ and that of *T*_leaf_ were also significantly correlated after both increases (*r*^2^ = 0.45) and decreases (*r*^2^ = 0.61) in light intensity. Our findings suggest that the times required for a change in Φ_PSII_ (particularly *t*_50_) and *T*_leaf_ (particularly *t*_90_) can be used as indicators of dynamic *A* and *g*_s_, respectively, facilitating faster phenotyping of the complex processes of photosynthesis and stomatal conductance kinetics in the future.

## Teaser

Rates of change in Φ_PSII_ and leaf temperature can be used as indicators of kinetics of CO_2_ assimilation rate and stomatal conductance for screening genotypic variation under fluctuating light.

## Introduction

Improving photosynthesis has been identified as a promising strategy for increasing crop productivity and ensuring future food security (Zhu *et al*., 2010; Long *et al*., 2015; Croce *et al*., 2024). One avenue to achieve this may be to breed for crops that respond more rapidly to fluctuations in light intensity (Lawson *et al*., 2012; Long *et al*., 2022). Focusing solely on steady-state photosynthesis is not representative of real-world conditions, as it overlooks the dynamic response of plants to their environment (Soleh *et al*., 2017, 2018; Salter *et al*., 2019; Acevedo-Siaca *et al*., 2020; Acevedo-Siaca and McAusland, 2025). Plants in the field and in greenhouses continuously experience dynamic conditions, including fluctuations in light intensity that are caused by changes in the solar angle and wind-induced movement of clouds, canopies, and individual leaves (Kaiser *et al*., 2018; Durand *et al*., 2022; van Westreenen *et al*., 2023). Since photosynthesis is a complex process and highly sensitive to environmental changes, the responses of several key photosynthesis-related processes to fluctuating light intensity tend to lag behind, generating an implicit inefficiency in photosynthetic performance (Slattery *et al*., 2018). For instance, slow responses by leaf photosynthesis to fluctuations in light intensity have been estimated to cause 10–40% losses of time-integrated photosynthesis per day, compared with a theoretically instantaneous response of net CO_2_ assimilation rate (*A*) to light intensity changes (Taylor and Long, 2017; Tanaka *et al*., 2019; Long *et al*., 2022). However, while non-steady-state photosynthesis is now becoming widely studied, rapid phenotyping methods for its properties are still lacking, unlike several tools that have become available for fast phenotyping of steady-state photosynthesis (Fu *et al*., 2022; Sarić *et al*., 2022; Acevedo-Siaca and McAusland, 2025). This hampers breeding efforts for faster kinetics of *A* and stomatal conductance (*g*_s_).

When a leaf in low light is suddenly exposed to a higher light intensity, *A* increases near-logarithmically until reaching a new steady state. This process, called photosynthetic induction, can take from a couple to several tens of minutes and is dependent upon genotype, the duration and intensity of light exposure, and other environmental conditions (Kursar and Coley, 1993; Kaiser *et al*., 2017b; Long *et al*., 2022; Acevedo-Siaca and McAusland, 2025). The speed of this change is often characterized by the time to reach a certain fraction of the steady state, for example the time to reach 50% induction (*t*_50_). The delay in *A* during photosynthetic induction is attributed to several transient biochemical and diffusional limitations. These limitations include the activation of Rubisco, the light-dependent activation of other Calvin-Benson-Bassham (CBB) cycle enzymes, as well as slow changes in mesophyll and stomatal conductance (Pearcy, 1990; Kaiser *et al*., 2015; Liu *et al*., 2022).

Stomatal conductance describes the rate of gaseous diffusion through small pores called stomata located on the leaf surface (Lawson, 2009). Utilizing a pair of specialized cells called guard cells, stomata actively regulate their aperture to balance carbon uptake with water loss. The speed with which these pores open or close affects both CO_2_ uptake and intrinsic water use efficiency (*A*/g_s_ = iWUE). Furthermore, stomatal opening and closing depend on various environmental factors, including light intensity and spectrum, air humidity, and temperature, as well as internal signals such as abscisic acid and other hormones (Lawson, 2009; Kaiser *et al*., 2019; Matthews and Lawson, 2019; Clark *et al*., 2022; Lawson and Leakey, 2024). The speed of photosynthetic induction is also affected by *g*_s_ which transiently limits the amount of CO_2_ available for assimilation (Kaiser *et al*., 2017b; Tanaka *et al*., 2019; Sakoda *et al*., 2022). Generally, stomata open under high light intensity, concurrently cooling the leaf via water vapor loss through transpiration. Conversely, stomata close under low light or darkness to limit water loss (Lawson and Blatt, 2014; Wall *et al*., 2023). However, neither of these responses is instantaneous, with stomata requiring several tens of minutes to fully open or close (Drake *et al*., 2013; McAusland *et al*., 2016).

Previous studies have documented extensive natural variation in non-steady-state *A* and *g*_s_ among crops such as rice (Taniyoshi *et al*., 2020; Acevedo-Siaca *et al*., 2020, 2021a), wheat (Salter *et al*., 2019), and soybean (Soleh *et al*., 2017). Given this diversity, it has been suggested that capturing variation for both *A* and *g*_s_ through large-scale phenotyping would be useful for identifying genotypes with potentially greater water and light use efficiency under fluctuating light conditions, as well as identifying an underlying genetic mechanism (McAusland *et al*., 2016; Soleh *et al*., 2017; Xiong *et al*., 2018; Salter *et al*., 2019; Tanaka *et al*., 2019; Acevedo-Siaca *et al*., 2020, 2021a, b; Pignon *et al*., 2021; N. Zhang *et al*., 2022, 2024; Croce *et al*., 2024; Acevedo-Siaca and McAusland, 2025). However, achieving this goal requires the development of quick and efficient methods to quantify variability in plant responses to light intensity fluctuations (Zhang *et al*., 2022; Wall *et al*., 2023). Leaf-level gas exchange utilizing infrared gas analysis is the benchmark for quantifying the kinetics of important photosynthetic traits during fluctuations in light. This method produces highly accurate values; however, it can be time-consuming as *A* and *g*_s_ often need to reach steady states before changing the light intensity for accurate measurements of photosynthetic induction and can be captured only in one leaf at a time. Therefore, protocols often exceed 30 min per sample, excluding the time required for adaptation to darkness or low light (Acevedo-Siaca and McAusland, 2025). High-throughput phenotyping methods such as chlorophyll fluorescence (CF) and thermal imaging may offer a solution to identify variations in photosynthetic performance across multiple crops or genotypes, simultaneously and repeatedly throughout plant growth (Baker, 2008; McAusland *et al*., 2013; Fu *et al*., 2022). However, it is currently unclear whether high-throughput phenotyping techniques can be used to quantify non-steady-state responses of *A* and *g*_s_ to fluctuations in light intensity.

The measurement of CF allows for the estimation of the operating efficiency of PSII (Φ_PSII_), which can be used to estimate linear electron flow during the light reactions of photosynthesis (Genty *et al*., 1989; Baker, 2008; Murchie and Lawson, 2013). Under steady-state conditions and in the absence of photorespiration, Φ_PSII_ is significantly correlated with *A* (Genty *et al*., 1989). However, greater effort needs to be made to characterize the relationship between these two parameters, especially under potentially stressful conditions such as during transitions to high light intensities. During photosynthetic induction, Φ_PSII_ increases slowly until reaching a steady state, similarly to *A* (Baker, 2008). The delayed response of photosynthesis during low to high light changes is due to slow activation of CBB cycle enzymes and opening of stomata. Previous work has indicated that *A* and Φ_PSII_ are often near-linearly related during photosynthetic induction (Morison *et al*., 2005; Kaiser *et al*., 2017b). However, it remains to be shown how well the time required for a change in Φ_PSII_ can be used to infer the time required for a change in *A*. CF can also elucidate how energy is being partitioned between the competing processes of photochemistry and photoprotection (Baker, 2008). Increased attention has been paid to characterizing the induction and relaxation kinetics of non-photochemical quenching (NPQ) in recent years, as researchers hypothesize that balancing photochemistry and photoprotection may lead to an optimization of photosynthetic performance (Murchie and Ruban, 2020). Ideally, there would be sufficient activation of NPQ to prevent photodamage and the production of reactive oxygen species (ROS) while avoiding excessive NPQ that would divert energy unnecessarily away from photochemistry. This balance between sufficient and excessive NPQ is especially relevant during transitions in light intensity, where NPQ can deploy too slowly or overshoot during low-to-high light transitions, or relax too slowly during high-to-low light transitions (Zhu *et al*., 2004). Consequently, examining the impact of NPQ on the kinetics of *A* and Φ_PSII_ during transitions in light intensity may also be informative.

In addition to utilizing CF to understand CO_2_ assimilation, thermal imaging can be used for non-contact measurements of leaf temperature (*T*_leaf_), and its alterations can be indicative of changes in the rate of transpiration, providing valuable information about stomatal opening and closure (Wang *et al*., 2004; McAusland *et al*., 2013; Vialet-Chabrand and Lawson, 2019, 2020; Zhang *et al*., 2025). Correlations between *g*_s_ as estimated from thermal imaging and *g*_s_ measured with leaf gas exchange have been documented previously (McAusland *et al*., 2013; Vialet-Chabrand and Lawson, 2019; Pignon *et al*., 2021). However, it is unclear whether the time required for a change in *T*_leaf_ can be used to reliably inform the time required for a change in *g*_s_ during fluctuations in light.

To our knowledge, no rapid methods for screening of non-steady-state responses of *A* and *g*_s_ to fluctuations in light intensity exist. As a first step to establishing such methods, we tested correlations between conventionally measured gas exchange-derived parameters and those based on CF and *T*_leaf_ under fluctuating light conditions. The objectives of this study were to (i) evaluate indicators of non-steady-state *A* and *g*_s_ by using Φ_PSII_ and *T*_leaf_; (ii) quantify the variation of these traits across nine species; and (iii) examine the relationship between steady-state and non-steady-state photosynthetic parameters among species.

## Materials and methods

### Plant material and growth conditions

Eight crop species and the model species *Arabidopsis thaliana* (Table 1) were grown in a climate chamber under controlled environmental conditions that were set to: 400 ppm CO_2_, average relative humidity of 70%, and a day/night temperature of 23/20 °C.

**Table 1.** Details of species used in the study, including Latin names and accession/cultivar name, plant age (number of days from sowing to measurement), and choice of the fully expanded, unshaded leaf used for measurements

Species	Name	Plant age	Leaf used for measurements
**Arabidopsis**	*Arabidopsis thaliana* Col-0	37	Fully mature unshaded leaf
**Basil**	*Ocimum basilicum* cv. Salvo*^a^*	33	Third from bottom
**Cucumber**	*Cucumis sativus* cv. Proloog*^b^*	28	Third from bottom
**French bean**	*Phaseolus vulgaris* cv. Modesto*^c^*	25	Second from bottom
**Lettuce**	*Lactuca sativa* cv. Jagger*^d^*	32	Fully mature unshaded leaf
**Strawberry**	*Fragaria×ananassa* cv. Elsanta*^e^*	∼35	Fully mature unshaded leaf
**Sweet pepper**	*Capsicum annuum* cv. Gialte*^f^*	31	Third from bottom
**Tomato**	*Solanum lycopersicum* cv. Foundation*^g^*	28	Second from bottom
**Wheat**	*Triticum aestivum* cv. Nobless *^h^*	23	Third from top on the main stem

Provided by: *^a^* CN seed, UK; *^b^* Rijk Zwaan, the Netherlands; *^c^* Van Hemert & Co, the Netherlands; *^d^* Nunhems Netherlands BV, the Netherlands; *^e^* Fresh Forward, the Netherlands; *^f^* Enza Zaden, the Netherlands; *^g^* BASF Nunhems, the Netherlands; *^h^* Limagrain, the Netherlands.

Lettuce seeds were vernalized in a darkroom at 4 °C for 72 h. Seeds of lettuce, cucumber, tomato, sweet pepper, basil, and French bean were germinated in rockwool plugs and subsequently transplanted into 10×10 cm rockwool cubes (Grodan, Roermond, the Netherlands). Wheat and strawberry plants were grown in soil (Lensli, Bleiswijk, the Netherlands) in plastic pots (11×11×12 cm). Strawberry plants were grown from runners. Photosynthetic photon flux density (PPFD) at canopy height was maintained at ∼190±10 µmol m^−2^ s^−1^ for 16 h (from 08.00 h to 24.00 h) with white LEDs (GreenPower LED toplighting compact, Philips, Signify Netherlands B.V., Eindhoven, the Netherlands). Nutrient solution with an electrical conductivity (EC) of 2.1 mS cm^−1^ and a pH of 5.5 was provided through an ebb and flow irrigation system twice per day.

*Arabidopsis thaliana* seeds (Col-0) were pre-sown in a Petri dish containing soaked filter paper, and placed in the darkroom at 4 °C for 72 h for vernalization. Seeds were then transferred to plastic pots (7×7×8 cm) containing soil (Lensli) and grown in the same climate chamber, but in a separate compartment with different light settings: Arabidopsis plants were grown under a 10 h photoperiod (from 08.00 h to 18.00 h) to avoid early flowering and small plant size (Gibeaut *et al*., 1997), which would make leaf gas exchange measurements difficult. PPFD was kept at ∼130±10 µmol m^−2^ s^−1^. Arabidopsis plants were irrigated twice a week with nutrient solution (EC ∼1.4 mS cm^−1^, pH ∼5.5). To reduce the impact of heterogeneous environmental factors, plants were rotated randomly twice per week.

### Gas exchange and chlorophyll fluorescence measurements

Gas exchange and CF were measured using the Li-6800 photosynthesis system (LI-COR Biosciences, Lincoln, NE, USA) equipped with the fluorometer leaf chamber (6800-01A, leaf area of 2 cm^2^) and leaf thermocouple. Measurements were conducted on fully expanded, unshaded leaves (Table 1) while main veins were avoided. In wheat, we adjusted the leaf area settings of the Li-6800 to account for narrow leaves. The light source of the fluorometer provided 90% red and 10% blue light (peak wavelengths: 625 nm and 475 nm, respectively). During measurements, conditions inside the cuvette were: 400 ppm CO_2_, 25 °C air temperature, 70% relative humidity, and 500 µmol s^−1^ air flow rate. Measurements were conducted between 09.00 h and 15.00 h, to minimize any diurnal effects on photosynthesis.

**Fig. 1.**
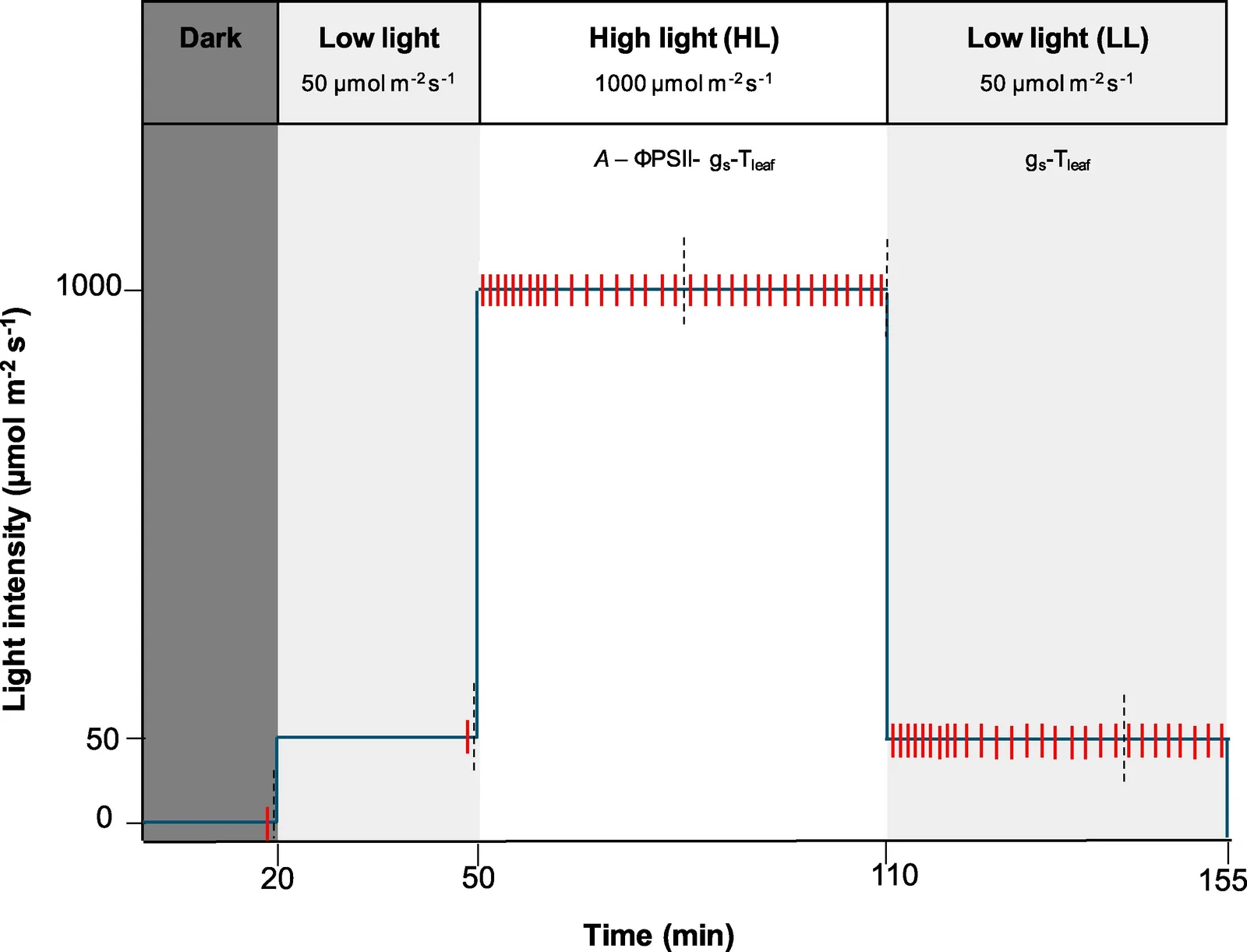
Overview of measurement protocol. The protocol consisted of four steps: (i) dark adaptation of the leaf for 20 min and application of a saturating pulse to measure chlorophyll fluorescence (CF) in the last minute; (ii) exposure to low light intensity (LL; 50 μmol m^−2^ s^−1^) for 30 min to stimulate activation of photosynthesis and stomatal opening, with CF parameters measured in the last minute; (iii) stepwise increase to high light (HL; 1000 μmol m^−2^ s^−1^) for 60 min; and (iv) stepwise decrease to LL for 45 min. During steps iii and iv, CF was measured every minute for the first 10 min, and every 2 min thereafter. The entire protocol lasted 155 min. Saturating pulses are shown as red vertical lines, measured parameters for steps iii and iv are displayed, and IRGA matching is shown as dashed black lines.

Each leaf was first dark-adapted in the Li-6800 cuvette for 20 min, after which minimum (*F*_o_) and maximum (*F*_m_) CF were recorded. Illumination was then increased to 50 µmol m^−2^ s^−1^ (low light—LL) for 30 min, to allow for stomatal opening and initial activation of photosynthesis. Afterwards, light intensity was increased to 1000 µmol m^−2^ s^−1^ (high light—HL) for 60 min, after which light intensity was reduced to LL again for another 45 min (Fig. 1).

One rectangular flash was applied in the final minute of the dark adaptation period to determine minimum and maximum CF fluorescence (*F*_o_ and *F*_m_, respectively). CF was measured once again at the end of the initial LL step through a 300 ms rectangular light pulse of 13000 µmol m^−2^ s^−1^ intensity. Subsequent saturating pulses were applied every minute during the first 10 min after a light intensity change, and every 2 min thereafter (Fig. 1, red lines) to determine maximum CF fluorescence under actinic light (*F*_m_′). Based on the dark modulation test (Li-Cor 6800 manual), CF measurement frequency was set to 100 Hz for all species, except basil and wheat, where 200 Hz provided a better signal-to-noise ratio. *A*, *g*_s_, and *T*_leaf_ were logged every 2 s. Infrared gas analyzers (IRGAs) were matched at the end of each light intensity step, as well as 30 min after a given switch in light intensity (Fig. 1, dashed black lines). Values of *A* and *g*_s_ were retrospectively corrected for discrepancies in measured H_2_O and CO_2_ concentrations due to IRGA drift during measurements. Saturating pulses caused sudden changes in heat load upon the leaf and thus artifacts in *g*_s_ and *T*_leaf_ (Supplementary Fig. S1); we therefore removed ∼22 s of data after each saturating pulse from *g*_s_ and *T*_leaf_ time courses (Kaiser *et al*., 2017b).

### Estimation of chlorophyll fluorescence parameters

Φ_PSII_ was calculated as in Genty *et al*. (1989): Φ_PSII_ = (*F*_m_′ − *F*′)/*F*_m_′; where *F*′ is fluorescence under actinic light. Maximum quantum efficiency of PSII photochemistry was calculated as: *F*_v_/*F*_m_ = (*F*_m_ − *F*_o_)/*F*_m_. NPQ was estimated as in Murchie and Lawson (2013): NPQ = (*F*_m_ − *F*_m_′)/*F*_m_′.

### Calculation of photosynthetic induction state and speed of response

To interpolate observed data points and obtain more accurate, noise-corrected estimates of time courses, a locally estimated scatterplot smoothing regression (LOESS) was fit to the data (Supplementary Fig. S2), using the function ‘loess’ from the ‘stats’ package in R (Cleveland *et al*., 1992; R Core Team, 2025). To fit this model on data, spans—degree of smoothing—were ∼5% for *A*, 15% for Φ_PSII_, and a range of 10–40% for *g*_s_ and *T*_leaf_, based on visual observation.

The induction state (IS) of photosynthesis was calculated as in Chazdon and Pearcy (1986): IS = (*A*_t_ − *A*_i_)/(*A*_f_ − *A*_i_) × 100, where *A*_t_ (µmol m^−2^ s^−1^) is the CO_2_ assimilation rate at time *t*; *A*_i_ (µmol m^−2^ s^−1^) is the initial rate of CO_2_ assimilation, and *A*_f_ (µmol m^−2^ s^−1^) is the final steady-state value of *A* reached under HL. For *A*_i_, the average of the last 2 min (24 data points) prior to changing to HL was used, while for *A*_f_ the average of the final 3 min (72 data points) under HL was used. The same method was applied to *g*_s_, *T*_leaf_, Φ_PSII_, and NPQ, with minor differences in calculating the initial values: for initial values of *A* and *g*_s_, the average of the previous steady state was used, while for *T*_leaf_, Φ_PSII_, and NPQ the first logged value after a switch to the new light intensity was used (Supplementary Fig. S2). Also, the differential value (Δ), as the final value minus the initial value, was calculated for each trait. Subsequently, the times to reach 50 (*t*_50_), 63.2 [time constant (τ)], and 90% (*t*_90_) of the full IS were estimated per trait based on the fitted curve.

### Statistical analysis

Per species, nine replicates were used for gas exchange measurements. Statistical tests for all parameters were performed in R (version 2024.12.0), using one-way ANOVA with a significance level of 0.05. To test for the normality of residuals, the Shapiro–Wilk test was used. If the assumption of normality was violated, the non-parametric Kruskal–Wallis test was used, which provided the same significance outcomes as ANOVA, indicating that normality of residuals did not influence the statistical significance of the outcomes. Tukey’s HSD test was then used to identify significant differences between species, using the ‘agricolae’ package (de Mendiburu, 2023). For simple linear regressions, the function ‘lm’ was used in R (R Core Team, 2025).

## Results

### Responses of photosynthetic traits to stepwise changes in light intensity

**Fig. 2.**
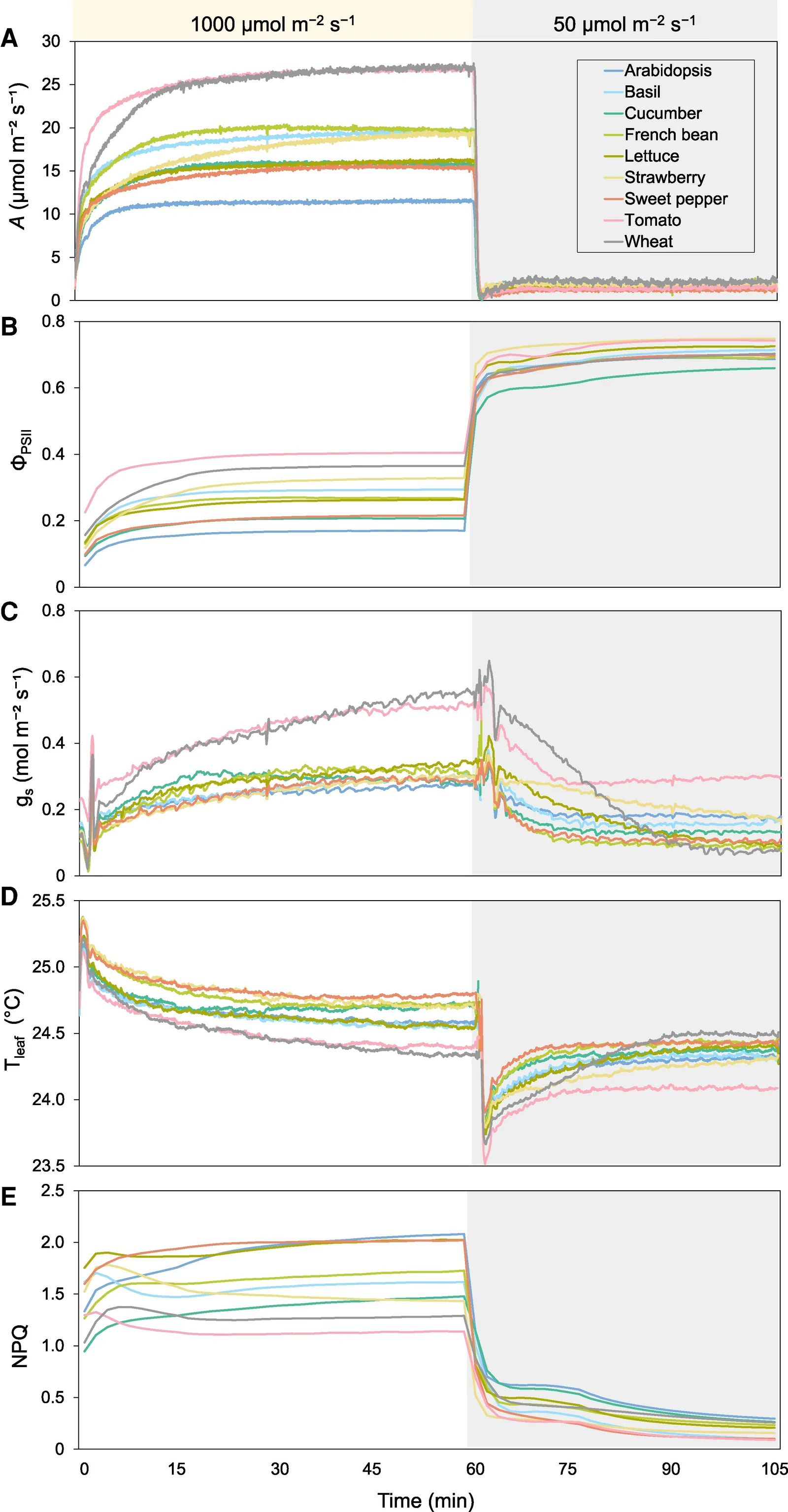
Kinetics of leaf gas exchange, chlorophyll fluorescence, and temperature in nine species under stepwise changes in light intensity. (A) Net photosynthesis rate (*A*), (B) PSII operating efficiency (Φ_PSII_), (C) stomatal conductance (*g*_s_), (D) leaf temperature (*T*_leaf_), and (E) non-photochemical quenching (NPQ). Light intensity was changed from 50 μmol m^−2^ s^−1^ to 1000 μmol m^−2^ s^−1^ for 60 min, followed by a decrease to 50 μmol m^−2^ s^−1^ for 45 min (gray shade). Curves display averages of nine replicates (*n* = 9) per species; error bars were omitted for greater visibility.

**Fig. 3.**
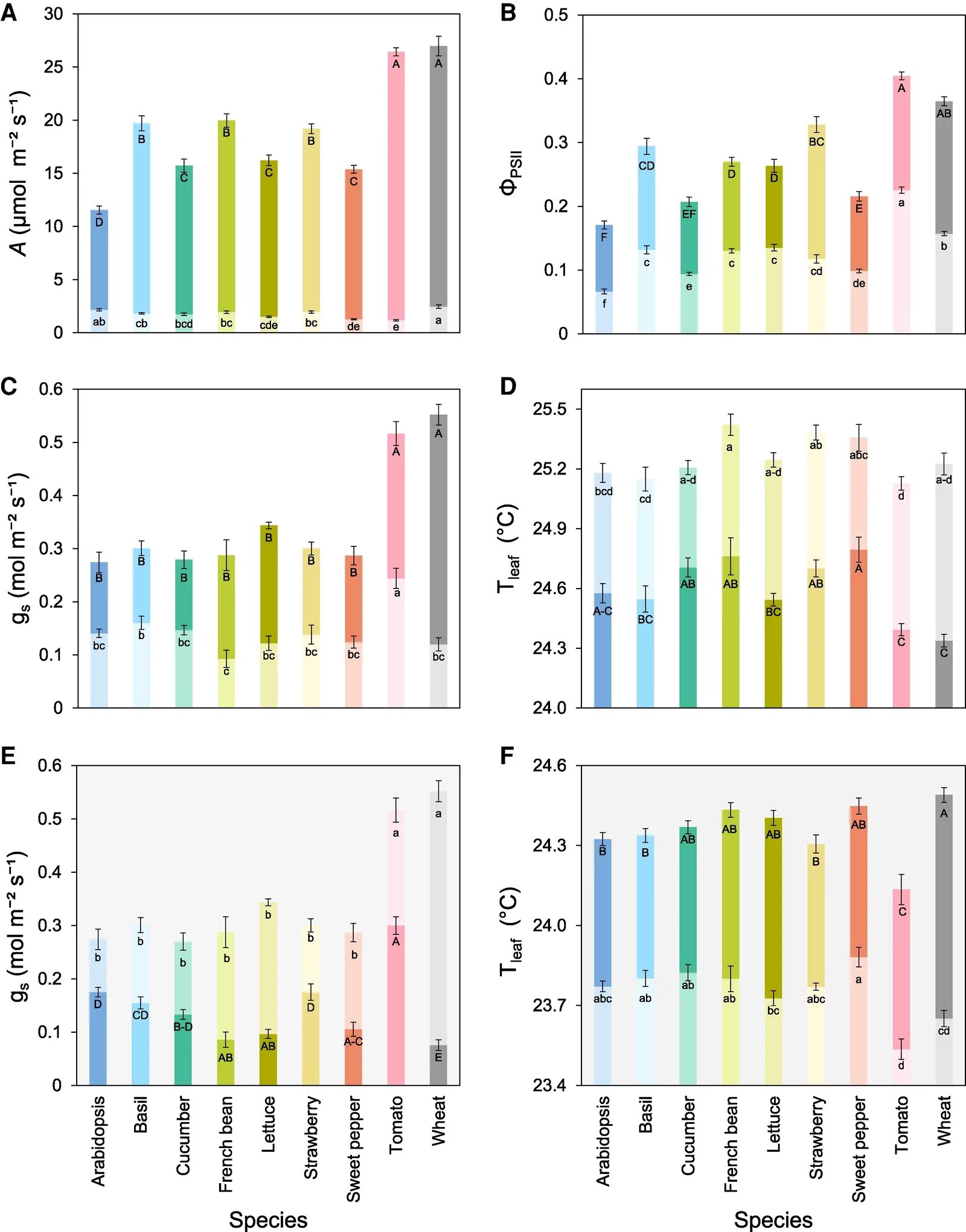
Steady-state values of leaf gas exchange, chlorophyll fluorescence, and temperature in nine species. (A) Net photosynthesis rate (*A*), (B) PSII operating efficiency (Φ_PSII_), (C and E) stomatal conductance (*g*_s_), and (D and F) leaf temperature (*T*_leaf_). Panels A–D show values related to the 50 μmol m^−2^ s^−1^ to 1000 μmol m^−2^ s^−1^ shift (H). Panels E and F (gray background) show values related to the 1000 μmol m^−2^ s^−1^ to 50 μmol m^−2^ s^−1^ shift (L). Per panel, lighter colors represent initial (i) and darker colors final values (f). Bars display means ±SE (*n* = 9). Lowercase letters represent significant differences among species for i, and uppercase letters for f (*P* < 0.05).

Following a 50 μmol m^−2^ s^−1^ (LL) to 1000 μmol m^−2^ s^−1^ (HL) increase in light intensity, both net CO_2_ assimilation rate (*A*) and Φ_PSII_ increased near-logarithmically in all species until reaching a steady state (Fig. 2A, B). Significant variation was observed for both initial and final steady-state, and differential values of *A* and Φ_PSII_ between species (*P* < 0.001 in all cases; Fig. 3A, B; Supplementary Fig. S3A, B). Values of initial *A* (*A*_i_) under LL ranged from 1.2 μmol m^−2^ s^−1^ to 2.5 µmol m^−2^ s^−1^, with tomato having the lowest and Arabidopsis and wheat having the highest values (Fig. 3A). Similarly, Arabidopsis had the lowest final steady-state value for *A* (*A*_f_; 11.5 µmol m^−2^ s^−1^), while wheat and tomato demonstrated the highest values (27.0 μmol m^−2^ s^−1^ and 26.4 µmol m^−2^ s^−1^, respectively; Fig. 3A). In regards to initial Φ_PSII_ (Φ_PSII,i_) under LL, Arabidopsis started at the lowest (0.07) and only reached a final Φ_PSII_ (Φ_PSII,f_) of 0.17 under HL, thus showing the lowest ΔΦ_PSII_ across species (Fig. 3B; Supplementary Fig. S3B). *A*_f_ and Φ_PSII,f_ were highest in wheat and tomato (0.37 and 0.4, respectively; Fig. 3B). As expected, upon reducing light intensity to 50 µmol m^−2^ s^−1^, *A* dropped quickly in all species, whereas Φ_PSII_ recovered more slowly and showed larger variability between species (Fig. 2A, B).

Both *g*_s_ and *T*_leaf_ displayed large and rapid initial responses due to stepwise changes in light intensity that affected leaf energy balance. Within several minutes of switching to HL, *g*_s_ showed slow increases, and declines when the light intensity was reduced back to LL (Fig. 2C). After an initial rise, *T*_leaf_ decreased slowly under HL, and increased slowly under LL (Fig. 2D), reflecting changes in transpiration rate due to stomatal opening and closure. Significant variation in *g*_s_ and *T*_leaf_ was identified among species for their initial, final, and differential values (*P* < 0.01, Figs 2C, D, 3C–F; Supplementary Fig. S3C–F). During photosynthetic induction, wheat and tomato demonstrated the highest *g*_s_ under HL, as quantified by larger *g*_s,f,H_ (0.55 mol m^−2^ s^−1^ and 0.52 mol m^−2^ s^−1^, respectively), and in wheat this increase caused a stronger reduction in *T*_leaf_ compared with the other species (Supplementary Fig. S3D). However, when transitioning to LL, tomato leaves experienced a smaller reduction in *g*_s_, and smaller changes in *T*_leaf_, while wheat displayed atypical behavior relative to other surveyed species, with a steeper change in *g*_s_ and *T*_leaf_, resulting in larger Δ*g*_s,L_ and Δ*T*_leaf,L_ (Fig. 2C, D; Supplementary Fig. S3E, F). This large change in wheat started from initial *g*_s_ under LL (*g*_s,i,L_ = 0.48 mol m^−2^ s^−1^) to its final steady state (*g*_s,f,L_ = 0.07 mol m^−2^ s^−1^) which was the lowest *g*_s,f,L_ among species; this was mirrored by the largest final *T*_leaf_, when transitioned to LL (*T*_leaf,f,L_) among all species (Fig. 3E, F).

NPQ exhibited significant differences in initial and final values across species upon transition to HL and relaxation upon transition to LL (*P* < 0.001 in all cases; Supplementary Fig. S4A–D), although differences between species were visually smaller during relaxation (Fig. 2E). Additionally, some species (tomato, wheat, basil, and strawberry) showed overshooting behavior during the activation of NPQ, reaching a temporary maximum NPQ after transitioning from LL to HL, and then settling into a final steady state (NPQ_f_,_H_) that was generally lower than the initial value in most of them (NPQ_i_,_H_; Fig. 2E). Arabidopsis, French bean, and sweet pepper showed the highest NPQ under 1000 µmol m^−2^ s^−1^ after 1 h (NPQ_f_,_H_ = 2.08, 2.2, and 2.2, respectively; Supplementary Fig. S4B), while basil, sweet pepper, and tomato reached the lowest values upon relaxation (NPQ_f,L_ = 0.1, 0.1, and 0.09, respectively; Supplementary Fig. S4B, D). All plants were unstressed, with *F*_v_/*F*_m_ ranging between 0.77 and 0.83 among the surveyed species (Supplementary Fig. S5).

### Speed of response to stepwise changes in light intensity

**Fig. 4.**
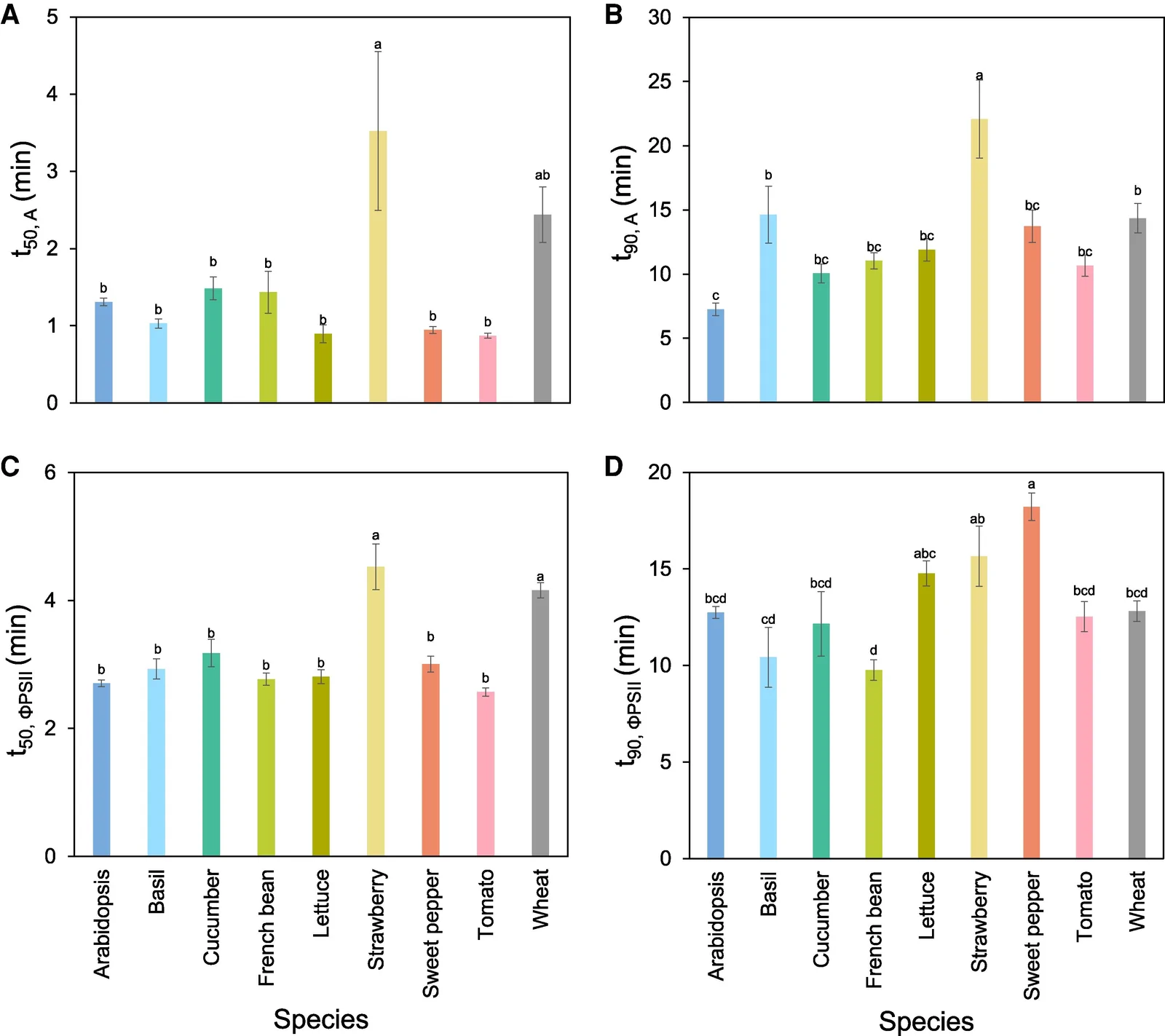
Response times of net photosynthesis rate (*A*) and PSII operating efficiency (Φ_PSII_) of nine species during photosynthetic induction. Panels show species-specific differences in time taken to reach 50% (*t*_50_; left panels) and 90% (*t*_90_; right panels) of the final steady-state value. (A, B) *A*, (C, D) Φ_PSII_ when light intensity was changed from 50 μmol m^−2^ s^−1^ to 1000 μmol m^−2^ s^−1^. Bars display means ±SE (*n* = 9); letters represent significant differences (*P* < 0.001).

**Fig. 5.**
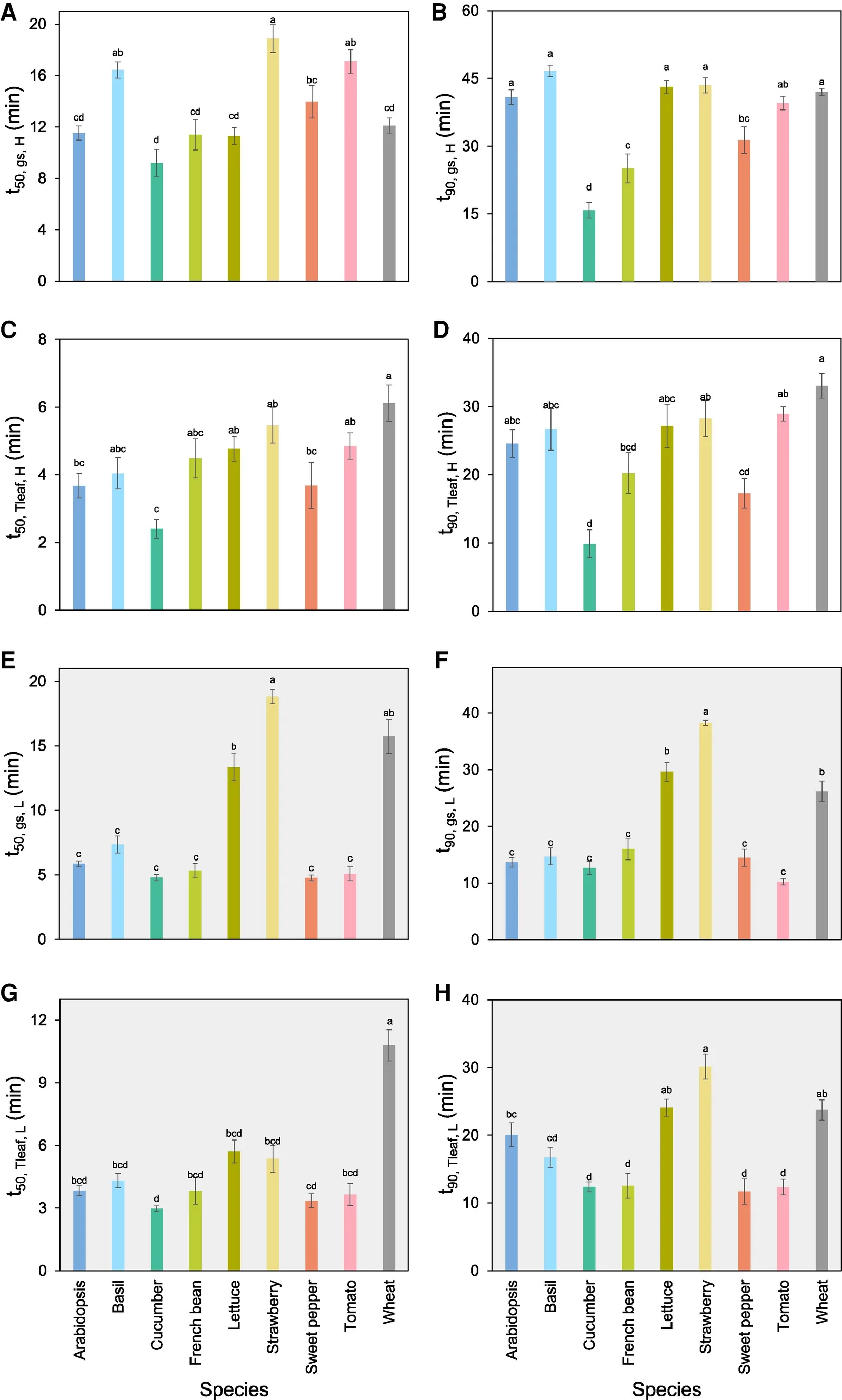
Response times of stomatal conductance (*g*_s_) and leaf temperature (*T*_leaf_) of nine species under stepwise changes in light intensity. Panels show species-specific differences in time taken to reach 50% (*t*_50_; left panels) and 90% (*t*_90_; right panels) of the final steady-state value. (A, B, E, and F) *g*_s_, and (C, D, G, and H) *T*_leaf_. Panels A–D Show values related to the 50 μmol m^−2^ s^−1^ to 1000 μmol m^−2^ s^−1^ shift (H). Panels E–H (gray background) show values related to the 1000 μmol m^−2^ s^−1^ to 50 μmol m^−2^ s^−1^ shift (L). Bars display means ± SE (*n* = 9); letters represent significant differences (*P* < 0.001).

The speed of response to increases in light intensity varied between species (*P* < 0.001; Figs 4A–D, 5A–D). The temporal range of both *t*_50,*A*_ and *t*_50, ΦPSII_ (0.9–4.5 min; Fig. 4A, C) was narrower than that observed for their *t*_90_ (7.3–22.1 min; Fig. 4B, D). The rate of induction in *A* and Φ_PSII_ was slower in strawberry and wheat, as demonstrated by large values for *t*_50,*A*_ (3.5 min and 2.4 min, respectively) as well as *t*_50, ΦPSII_ (4.5 min and 4.2 min, respectively; Fig. 4A, C). In strawberry, the time to reach 90% of steady state in *A* (*t*_90,*A*_) was 22.1 min, which was significantly (*P* < 0.001) slower than that of the other species (Fig. 4B). During photosynthetic induction, cucumber tended to have the shortest time to reach 90% of final *g*_s_ (*t*_90,*g*s,H_), indicating a faster increase in *g*_s_ and a corresponding decrease in *T*_leaf_ with 15.8 min and 9.9 min, respectively (Fig. 5B, D).

After the transition from high to low light intensity, species showed significantly different responses over time (*P* < 0.001; Fig. 5E–H; Supplementary Fig. S6). Strawberry and wheat showed the slowest decline in *g*_s_, as signified by the largest *t*_50,*g*s,L_ (18.8 and 15.7 min, respectively; Fig. 5E) and *t*_90,*g*s,L_ (38.3 min and 26.2 min, respectively; Fig. 5F). Similarly, *T*_leaf_ took longer for strawberry and wheat to reach *t*_90_ (30.1 min and 23.7 min, respectively; Fig. 5H). The rapidity of stomatal closure under LL was in some species greater than that of stomatal opening under HL: for instance, *t*_50,*g*s,H_ and *t*_50,*g*s,L_ for basil were 16.4 min and 7.3 min, respectively, while *t*_50,*g*s,H_ and *t*_50,*g*s,L_ were near-identical in strawberry (∼18.8 min; Fig. 5A, E). In regards to NPQ relaxation after the stepwise reduction in light intensity, this was fastest in sweet pepper (*t*_90,NPQ,L_ = 15.9 min), while there were only minor differences among the other species (Supplementary Fig. S6).

### Correlations of non-steady-state photosynthetic traits to stepwise changes in light intensity

**Fig. 6.**
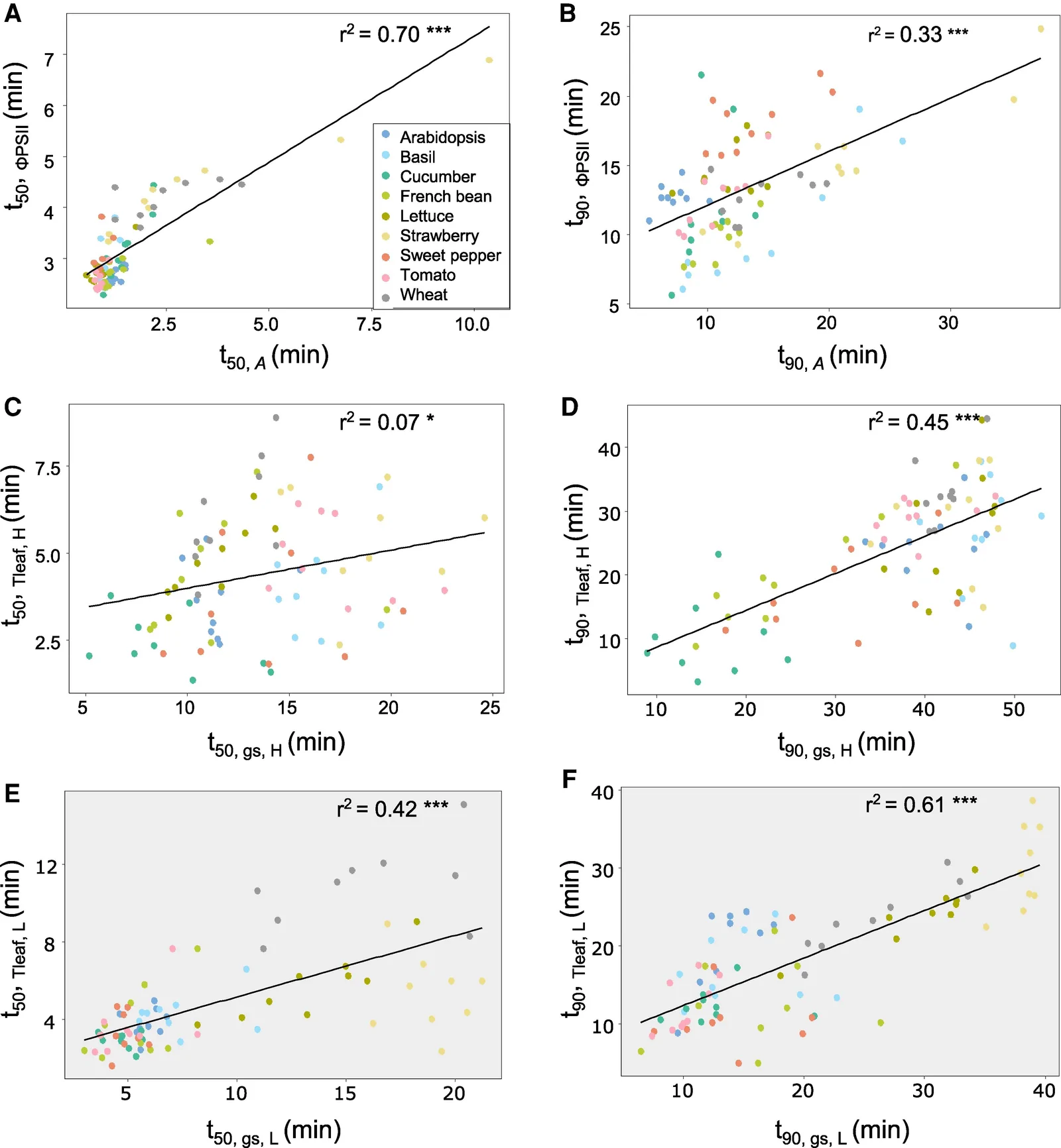
Rates of change of photosynthesis and stomatal conductance correlate with those of chlorophyll fluorescence and leaf temperature, respectively. Panels show correlations between times to reach 50% (*t*_50_; left panels) and 90% (*t*_90_; right panels) of the final value per given trait. (A, B) Times for net photosynthesis rate (*A*) versus PSII operating efficiency (Φ_PSII_) and (C, D) stomatal conductance (*g*_s_) versus leaf temperature (*T*_leaf_) when light intensity was changed from 50 μmol m^−2^ s^−1^ to 1000 μmol m^−2^ s^−1^ (H), and (E, F) *g*_s_ versus *T*_leaf_ when transitioning from 1000 μmol m^−2^ s^−1^ to 50 μmol m^−2^ s^−1^ (L; gray background). Each dot represents a biological replicate, with nine replicates per nine species (*n* = 81); associated *P*-values and *r*^2^ are shown per correlation. Asterisks indicate statistical significance levels: *P < 0.05, **P < 0.01, ***P < 0.001.

Across species, there was a strong linear correlation (*r*^2^ = 0.70, *P* < 0.001) between the time to reach 50% of final *A* (*t*_50,*A*_) and the time to reach 50% of final Φ_PSII_ (*t*_50, ΦPSII_; Fig. 6A). However, *t*_90,*A*_ and *t*_90, ΦPSII_ displayed a larger range of responses across species with a weaker correlation (*r*^2^ = 0.33, *P* < 0.001; Fig. 6B). A positive correlation (*r*^2^ = 0.72) was also found between the time constant of *A* (τ*_A_*) and that of Φ_PSII_ (τ_ΦPSII_) (*P* < 0.001; Supplementary Fig. S7).

During photosynthetic induction, there was a positive correlation (*r*^2^ = 0.45, *P* < 0.001) between *t*_90,*g*s,H_ and *t*_90,*T*leaf,H_, while the correlation between *t*_50,*g*s,H_ and *t*_50,*T*leaf,H_ was not as strong, though still statistically significant (*r*^2^ = 0.07, *P* < 0.05; Fig. 6C, D). On the other hand, when transitioning from HL to LL, positive correlations with *r*^2^ = 0.42 between *t*_50,*g*s,L_ and *t*_50,*T*leaf,L_, and *r*^2^ = 0.61 between *t*_90,*g*s,L_ and *t*_90,*T*leaf,L_ were observed (*P* < 0.001; Fig. 6E, F). Furthermore, τ*_g_*_s_ and τ*_T_*_leaf_ showed significant correlations under both light steps, with a much stronger correlation after the HL to LL shift compared with the LL to HL shift (*r*^2^ = 0.62 and *r*^2^ = 0.14, respectively; Supplementary Fig. S7B, C).

### Species-specific correlations of non-steady-state photosynthetic traits

Assessing the relationships between *A* and Φ_PSII_ response time per species (among the nine biological replicates per species) revealed consistently positive correlations across all species, but with different slopes, which were not always significant (Table 2; Supplementary Fig. S8). For example, the relationship between *t*_50,*A*_ and *t*_50, ΦPSII_ showed a range of correlation strengths, from non-significant in sweet pepper (*r*^2^ = 0.15) to significant in strawberry (*r*^2^ = 0.95, *P* < 0.01; Table 2; Supplementary Fig. S8). Non-steady-state *g*_s_ and *T*_leaf_ correlations per species spanned a range of negative, near-zero, and positive values for *t*_50_, while all correlations were positive for *t*_90_ except for basil under HL, which was close to zero (Supplementary Figs S9, S10; Table 2). Under both light steps at all three measured time points, some species, including lettuce and wheat, consistently showed a positive slope, which was significant in most cases (Supplementary Figs S9, S10). However, all negative *g*_s_–*T*_leaf_ correlations were found to be non-significant (Table 2). A larger number of significant *g*_s_–*T*_leaf_ correlations was observed upon switching from HL to LL (Supplementary Figs S9, S10). The statistical significance of correlations observed for *t*_50_ differed from that observed for *t*_90_ in paired traits. Lettuce showed the largest number of correlations (Table 2).

**Table 2.** Coefficient of determination (*r*^2^) per species

Species	*r* ^2^ (*A,* Φ_PSII_)				*r* ^2^ (*g*_s,_ *T*_leaf_)				*r* ^2^ (*g*_s,_ *T*_leaf_)			
	50→1000 μmol m^−2^ s^−1^				50→1000 μmol m^−2^ s^−1^				1000→50 μmol m^−2^ s^−1^			
	*t*_50_		*t*_90_		*t*_50_		*t*_90_		*t*_50_		*t*_90_	
Arabidopsis	0.38^ns^	+	0.08^ns^	+	<0.01^ns^	+	<0.01^ns^	+	0.04^ns^	+	0.55*	+
Basil	0.34^ns^	+	0.86**	+	0.10^ns^	+	<0.01^ns^	–	0.08^ns^	+	<0.01^ns^	+
Cucumber	0.86**	+	0.16^ns^	+	0.20^ns^	–	<0.01^ns^	+	0.07^ns^	–	0.01^ns^	+
French bean	0.66**	+	0.70**	+	0.01^ns^	+	0.92**	+	0.34^ns^	+	0.03^ns^	+
Lettuce	0.86**	+	0.20^ns^	+	0.74**	+	0.30^ns^	+	0.78**	+	0.86**	+
Strawberry	0.95**	+	0.89**	+	0.01^ns^	–	<0.01^ns^	+	0.04^ns^	–	0.33^ns^	+
Sweet pepper	0.15^ns^	+	0.50*	+	0.03^ns^	+	0.20^ns^	+	<0.01^ns^	+	0.07^ns^	+
Tomato	0.40^ns^	+	0.76**	+	0.17^ns^	–	0.20^ns^	+	0.25^ns^	+	0.43^ns^	+
Wheat	0.41^ns^	+	0.18^ns^	+	0.47*	+	0.33^ns^	+	0.21^ns^	+	0.82**	+

Times to reach 50% (*t*_50_) and 90% (*t*_90_) for net photosynthesis rate (*A*) versus PSII operating efficiency (Φ_PSII_) and stomatal conductance (*g*_s_) versus leaf temperature (*T*_leaf_) when light intensity was changed from 50 μmol m^−2^ s^−1^ to 1000 μmol m^−2^ s^−1^, and *g*_s_ versus *T*_leaf_ when transitioning from 1000 μmol m^−2^ s^−1^ to 50 μmol m^−2^ s^−1^. Direction of correlation is indicated as positive (+) and negative (−). Significant correlations are shown in bold. Asterisks indicate statistical significance levels: *P < 0.05, **P < 0.01; ns = not significant (*n* = 9).

### Correlations of steady-state and non-steady-state photosynthetic traits

**Fig. 7.**
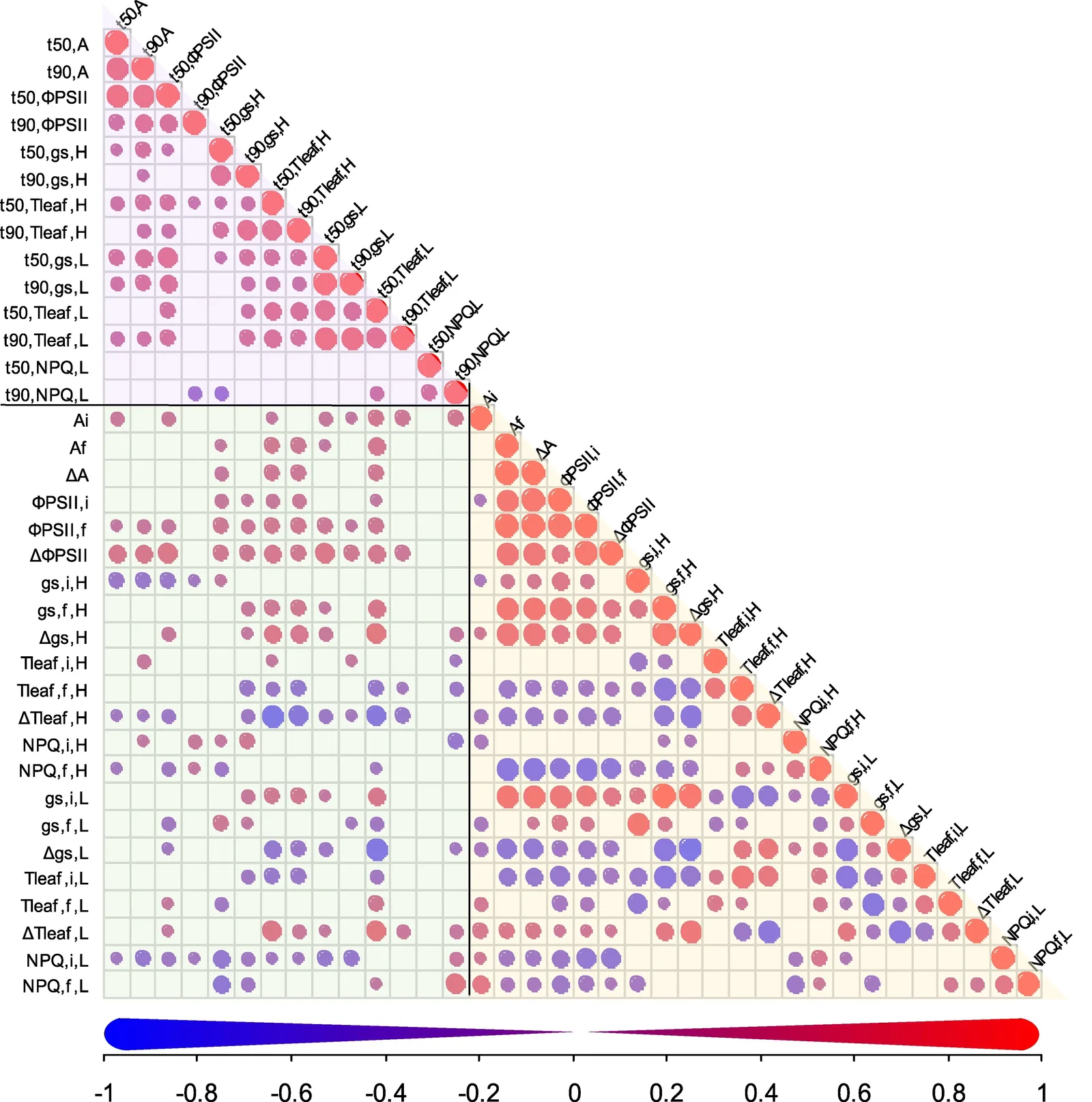
Correlation matrix of all measured traits, including those among steady-state (yellow shade, bottom right corner), those among non-steady-state traits (purple shade, upper left corner), and correlations between them (green shade, bottom left corner). Circles are present when the *P*-value is significant (*P* < 0.05); the size of circles indicates the strength of the correlation coefficient (*r*). Positive and negative correlations are shown in red and blue, respectively. Trait names are abbreviated as follows: net photosynthesis rate (*A*), PSII operating efficiency (Φ_PSII_), stomatal conductance (*g*_s_), leaf temperature (*T*_leaf_), non-photochemical quenching (NPQ), time to reach 50% (*t*_50_) and 90% (*t*_90_) of final steady-state, light intensity of 1000 μmol m^−2^ s^−1^ (H) and 50 μmol m^−2^ s^−1^ (L), and differences (Δ) between the initial (i) and final (f) value.

A correlation matrix across all measured traits revealed a relatively larger number of linear correlations (*P* < 0.05) among steady-state (Fig. 7, yellow shade) and non-steady-state traits (Fig. 7, purple shade), but only few correlations between them (Fig. 7, green shade). Significant correlations were found between all final steady-state values, namely positive correlations between *A*_f_ and Φ_PSII,f_ (*r* = 0.91), and between *g*_s,f,H_ (*r* = 0.8), and NPQ_f,H_, and a negative correlation between *A*_f_ and *T*_leaf,f,H_ (*P* < 0.05 in all cases; Fig. 7). Also, during both light steps, species with higher final steady-state *g*_s_ had smaller *T*_leaf_ [negative correlation between *g*_s,f,H_ and *T*_leaf,f,H_ (*r* = −0.75); *g*_s,f,L_ and *T*_leaf,f,L_ (*r* = −0.82)] and smaller NPQ (negative correlation of *g*_s,f,H_ and NPQ_,f,H_; *g*_s,f,L_ and NPQ_,f,L_; *P* < 0.05 in all cases). Some steady-state traits also correlated with non-steady-state traits (e.g. NPQ_f,L_ and *t*_90,NPQ,L_, *g*_s,i,H_, and *t*_50,A_ as well as *t*_50, ΦPSII_; Fig. 7) while in most cases the response time of a given trait did not correspond to its steady-state value; for instance, there was no significant correlation between *t*_50,*A*_ and *A*_f_ or *g*_s,i_ and *t*_90,*g*s_ (Fig. 7).

We found significant correlations for times to reach final steady-state *A* with those for *g*_s_ and *T*_leaf_ for both *t*_50_ and *t*_90_. As an example, there were significant correlations of *t*_50,*A*_ with *t*_50,*g*s,H_ and *t*_50,*T*leaf,H_ (*r* = 0.3 for both; Fig. 7). The response times (*t*_50_ and *t*_90_) of almost all traits were consistently positively correlated with one another. As an exception to this, *t*_50,NPQ,L_ did not show any significant correlation with any measured trait, except for *t*_90,NPQ,L_. Furthermore, the time for stomatal opening under HL (*t*_50,*g*s,H_ and *t*_90,*g*s,H_) was correlated with the time for stomatal closing under LL (*t*_50,*g*s,L_ and *t*_90,*g*s,L_, respectively; *P* < 0.05). This pattern was also observed in the leaf temperature response (Fig. 7).

## Discussion

The kinetics of leaf net CO_2_ assimilation (*A*) and stomatal conductance (*g*_s_) under fluctuating light intensity are seen as important traits that underpin plant growth, but measuring them is currently time-consuming, thus hampering their screening in larger plant populations. We found significant relationships among gas exchange parameters of non-steady-state *A* and *g*_s_ with Φ_PSII_ and *T*_leaf_, which may be used in high-throughput phenotyping methods in the future.

### Non-steady-state *A* and Φ_PSII_ are significantly correlated during photosynthetic induction

Previous studies have documented relationships between steady-state *A* and Φ_PSII_ across light intensities and CO_2_ concentrations (Genty *et al*., 1989; Morison *et al*., 2005; McAusland *et al*., 2013), as well as non-steady-state *A* and electron transport rate (ETR) during photosynthetic induction under diverse environmental conditions (Yamori *et al*., 2012; Kaiser *et al*., 2017a), indicating that *A* and Φ_PSII_ are linked under diverse environmental scenarios. Given similar responses of *A* and Φ_PSII_ during photosynthetic induction (Fig. 2; Kaiser *et al*., 2017a, 2020), and under fluctuating light (Vialet-Chabrand *et al*., 2017), a correlation between their rate of response to a step change in light intensity was hypothesized and experimentally confirmed in this study. To our knowledge, ours is the first study to show that this relationship exists.

In addition to the strong correlations between steady-state *A* and Φ_PSII_ (Fig. 7), we observed significant correlations between their non-steady-state values at three time points (*t*_50_, τ, and *t*_90_) during photosynthetic induction (Fig. 6A, B; Supplementary Fig. S7A). A narrower time range and a stronger correlation for *t*_50_ (*r*^2^ = 0.7) compared with *t*_90_ (*r*^2^ = 0.33; Figs 4A–D, 6A, B) suggests that most species respond rapidly and comparably uniformly during the initial phase of induction, but show greater variability when approaching steady-state values. This could be advantageous under natural conditions, where light intensity fluctuates rapidly (Durand and Robson, 2023), and leaf photosynthesis may be closer to *t*_50_ than to *t*_90_ when responding to these frequent sunflecks. Although correlation strength varied across induction phases and species (Table 2; Supplementary Fig. S8), it was consistently positive, providing strong evidence for a coordinated rapidity of response. Accordingly, a faster rise in *A* generally correlated with a faster rise in Φ_PSII_. This supports the notion that CF imaging could be used to screen for variation of photosynthetic induction rate in large populations. A possible breeding target for photosynthetic improvement in this context could be *t*_50, ΦPSII_, which was a more reliable indicator of *t*_50,*A*_ than *t*_90, ΦPSII_ was for *t*_90,*A*_ (Fig. 6).

However, further efforts need to be made to understand if the relationship found in this study applies under stressful conditions, such as drought and excessive heat. Currently, there is a significant gap in our understanding of photosynthetic induction and its related limitations within the context of stressful environmental conditions. It could be expected that the relationship between the time constants of CO_2_ assimilation and Φ_PSII_ may become uncoupled under conditions of stress. This could be due to more electrons finding alternative sinks during the light reactions, such as the formation of ROS or cyclic electron flow (Yamori and Shikanai, 2016), as well as the products of linear electron flow being used towards other processes such as photorespiration, which can also increase during periods of environmental stress (Walker *et al*., 2016). As such, more attention needs to be given to characterizing photosynthetic induction—and photosynthetic performance under fluctuating light in general—under stress conditions and evaluating if the relationship between CO_2_ assimilation and Φ_PSII_ presented here remains.

### Non-steady-state *g*_s_ and *T*_leaf_ are significantly correlated under stepwise changes in light intensity

Stomatal opening progressively lowers *T*_leaf_ by increasing transpiratory cooling, assuming all other factors remain constant. Conversely, stomatal closure increases *T*_leaf_ by restricting leaf transpiration (Fig. 2C, D; McAusland *et al*., 2013). This relationship resulted in the expected negative correlation between steady-state *g*_s_ and T_leaf_ (Fig. 7). Pignon *et al*. (2021) reported a correlation (*r*^2^ = 0.30) between the time to reach minimum *g*_s_ measured using leaf gas exchange, and that estimated from thermal imaging in 64 *Sorghum bicolor* genotypes after a switch from high to low light intensity. In the current study, the *r*^2^ of *t*_90_ in *T*_leaf_ and *g*_s_ after a stepwise reduction in light intensity was twice as high (*r*^2^ = 0.61, Fig. 6F) as that reported by Pignon *et al*. (2021). Weak *g*_s_ and *T*_leaf_ correlations in earlier studies were attributed to separate thermal and gas exchange measurements, where external factors such as air temperature and airflow may have influenced *T*_leaf_ and thermal-imaging-based *g*_s_ estimation (McAusland *et al*., 2013; Pignon *et al*., 2021; Dupont *et al*., 2025) as well as to within-leaf heterogeneity in photosynthetic parameters such as *A*, *g*_s_, and *T*_leaf_ (Murchie and Lawson, 2013; Hõrak *et al*., 2021). A distinguishing element of the current study is that *g*_s_ and *T*_leaf_ were measured simultaneously on the same leaf, thus probably minimizing discrepancies between them, potentially leading to larger correlation coefficients for their non-steady-state values.

Our results showed a positive correlation between non-steady-state *g*_s_ and *T*_leaf_ after changing light intensity (*r*^2^ ranging from 0.07 to 0.62; Fig. 6C–F; Supplementary Fig. S7B, C), although this correlation was not consistently positive within each species (Table 2). In contrast to the relationship between *A* and Φ_PSII_, these correlations between *g*_s_ and *T*_leaf_ were stronger for *t*_90_ than for *t*_50_. Weaker correlations at *t*_50_, along with varying direction of correlation per species at this stage, are likely to reflect greater variability and species-specific delays in the early phase of their stomatal responses. This may be partly due to a smaller absolute amplitude in *T*_leaf_ during light transitions (∼0.5–0.9 °C; Supplementary Fig. S3D, F), as well as some variability in *g*_s_ and *T*_leaf_ data (Fig. 2C, D). Both of these factors may have placed *t*_50_ close to initial values and increased its sensitivity to noise, presenting a technical limitation that potentially affected the correlation analysis outcomes. In contrast, stronger correlations for *t*_90_ suggest a greater influence of *g*_s_ on *T*_leaf_ during the later phase of light transitions, probably due to a delay before this effect became apparent. Species-specific correlations (Table 2) highlight the need to investigate underlying mechanisms of transpiration and stomatal responses to light, as well as variations in leaf anatomy that lead to positive, negative, or neutral relationships. For future studies, we recommend using *t*_90,*T*leaf_ as an indicator of *t*_90,*g*s_ for screening of stomatal response kinetics to stepwise changes in light intensity, particularly when screening for the rate of stomatal closure (Fig. 6E, F).

### Significant natural variation exists between species for photosynthetic traits under fluctuating light

A large diversity in the traits that regulate photosynthetic responses to changes in light intensity was previously reported between species (McAusland *et al*., 2016; Zhang *et al*., 2022, 2024) and genotypes (Soleh *et al*., 2017; Salter *et al*., 2019; Acevedo-Siaca *et al*., 2020, 2021b). Surveyed species showed considerable variation across all steady- and non-steady-state traits (Figs 2–5; Supplementary Figs S3–S6), with observed correlations differing among species (Table 2; Supplementary Figs S8–10), reflecting species-specific photosynthetic regulations, strategies, and limitations in response to light transitions (Zhang *et al*., 2022; Suwannarut *et al*., 2023; Acevedo-Siaca and McAusland, 2025).

Photosynthetic traits can be influenced by leaf biochemistry and anatomy, including that of the stomata (McAusland *et al*., 2013). Stomatal behavior depends on several structural characteristics, including stomatal size, density, dimension, and shape (Faralli *et al*., 2019; Gray *et al*., 2020), guard cell physiology, and biochemistry (Lawson and Blatt, 2014; Pignon *et al*., 2021). All these traits show spatial heterogeneity not only within a single leaf (Vialet-Chabrand and Lawson, 2020) but also between its surfaces (Wall *et al*., 2022). Some species can adjust *g*_s_ to light changes faster due to the mentioned stomatal characteristics. For example, in this study, cucumber leaves showed the quickest response, followed by faster changes in *T*_leaf_ (Fig. 5A–H), probably due to the higher stomatal density and small stomatal size seen in cucumber (Zhang *et al*., 2022).

Furthermore, it is extensively documented that stomatal size, distribution, patterning, and whether stomata are sunken or raised directly influence the boundary layer around the surface of the leaf (Gray *et al*., 2020). These factors, which were not accounted for directly in this study, would almost certainly affect the relationship between *g*_s_ and *T*_leaf_ under field or greenhouse conditions. One of the limiting factors of this study was that leaf temperature was measured concurrently with gas exchange parameters, in the absence of a boundary layer. While we think that the approach presented here allows for the capture of larger differences between species, under more variable conditions and with the presence of the boundary layer, it may be more difficult to detect subtle differences. In this case, it would also be important to characterize (and account for) the microclimate around the leaf as well as the stomatal anatomy or patterning that could influence the boundary layer. Consequently, it would be of interest to further examine the specific relationship between species-dependent stomatal anatomical traits and the kinetics of photosynthetic induction, its underlying limitations, and its sensitivity to environmental cues.

### Few correlations exist between non-steady-state and steady-state parameters

Our results showed limited correlations between steady-state and non-steady-state photosynthetic traits, while their correlations within each category were consistently stronger (Fig. 7). For instance, steady-state *A*_f_ showed significant correlations with final *g*_s_, Φ_PSII_, *T*_leaf_, and NPQ values under HL, while a relationship between *t*_50,*A*_ and *A*_f_ was absent (Fig. 7). This supports work from an earlier study in rice (Acevedo-Siaca *et al*., 2020), highlighting that steady-state measurements may overlook important aspects of plant responses to environmental changes (Soleh *et al*., 2017; Salter *et al*., 2019; Acevedo-Siaca *et al*., 2020; Long *et al*., 2022; Shao *et al*., 2024; Acevedo-Siaca and McAusland, 2025). Limitations and mechanisms underlying steady-state and non-steady-state photosynthetic traits may differ, emphasizing the necessity of further investigation using faster phenotyping methods or suitable proxies. Greater variation reported in non-steady-state compared with steady-state traits (Acevedo-Siaca *et al*., 2020; Zhang *et al*., 2022) presents a promising opportunity to study and improve photosynthetic efficiency under fluctuating light.

Furthermore, our results support previous observations that the response rate is independent of the differences between the two steady-state values (McAusland *et al*., 2016). For instance, strawberry had the slowest response for most traits, but it did not generally exhibit the largest amplitudes between steady states (Figs 3–5). Thus, species with similar steady states or differential values did not necessarily show similar rates of response, as reflected by weak or absent correlations in Fig. 7. A fast response coupled with high steady state can be advantageous; for example, this combination in *g*_s_ can reduce diffusional limitations on *A* (McAusland *et al*., 2016).

Genotypic variation in stomatal opening can contribute to variation in photosynthetic induction (Deans *et al*., 2019; Taniyoshi *et al*., 2020), with faster changes in *g*_s_ leading to a faster rate of induction and thus less forgone assimilation in some species or genotypes (Deans *et al*., 2019; Shimadzu *et al*., 2019; Yamori *et al*., 2020; Zhang *et al*., 2022). However, this relationship is not always observed under dynamic conditions (Knapp and Smith, 1987; McAusland *et al*., 2013). In our study, *t*_50,*A*_ and *t*_50,*g*s,H_ showed a smaller strength of correlation (*r* = 0.3) than their respective steady states (*A*_f_ and *g*_s,f_; *r* = 0.8; Fig. 7). Transient *g*_s_ does not necessarily affect the entire period of photosynthetic induction (Zhang *et al*., 2022); instead, it often co-limits photosynthetic induction, together with biochemical limitations and mesophyll conductance (Kaiser *et al*., 2015; Acevedo-Siaca *et al.*, 2021a; Liu *et al*., 2022). Similar to earlier studies (Lawson and Blatt, 2014; McAusland *et al*., 2016), stomatal responses occurred much more slowly and varied more than changes in *A* (Fig. 4A, C). However, the primary limitation(s) of photosynthetic induction remain under debate, with different studies identifying different key constraints, as the extent to which each limitation acts on photosynthetic induction is highly context dependent (Vialet-Chabrand *et al*., 2024).

Significant correlations under both light steps between *g*_s,i_ and *t*_50,*g*s_, but not *t*_90,*g*s_ (Fig. 7), indicate that leaves with larger *g*_s,i_ required more time to further open or close their stomata (Lawson and Blatt, 2014; McAusland *et al*., 2016; Pignon *et al*., 2021). Higher *g*_s,i,H_ shortened the response time of *A* and Φ_PSII_ induction (Fig. 7; Kaiser *et al*., 2016, 2020; Zhang *et al*., 2022), probably due to more CO_2_ being available for CO_2_ assimilation (Kaiser *et al*., 2020). In addition, faster stomatal opening was typically coupled with faster closure, as well as quicker *T*_leaf_ changes after both light steps (Fig. 7), for example with cucumber consistently showing fast and strawberry showing slow *g*_s_ changes (Fig. 5). Consistent with previous reports, this coordination occurs regardless of guard cell shape (elliptical or dumbbell shaped), indicating shared mechanisms controlling stomatal movement (McAusland *et al*., 2016; Pignon *et al*., 2021). In some cases, the rate of stomatal closure was previously reported to be faster than stomatal opening, leading to enhanced iWUE and acting as a desirable potential trait for selection (McAusland *et al*., 2016; Vialet-Chabrand and Lawson, 2019). This pattern was observed in most species, with a few exceptions, such as *t*_90_ in strawberry (Fig. 5A–H).

Considering the recent focus on NPQ relaxation (Kromdijk *et al*., 2016; De Souza *et al*., 2022; Kromdijk and Walter, 2023), we examined how NPQ relaxation is related to other measured photosynthetic parameters. Our work revealed only a few significant relationships between *t*_90,NPQ,L_ and other traits, and none for *t*_50,NPQ,L_ (Fig. 7). Furthermore, the rate of NPQ relaxation showed little or no relationship with other steady- and non-steady-state traits, suggesting potential for independent improvement or selection relative to other photosynthetic traits. However, steady-state values for initial NPQ during low-light conditions correlated significantly with the time to 50% and 90% of full induction for *A*, Φ_PSII_, *g*_s_, and *T*_leaf_ (Fig. 7). This aligns with previous studies that have shown that having lower levels of accumulated photoprotection during low-light conditions may be beneficial upon transitions to high-light conditions (Kromdijk *et al*., 2016; De Souza *et al*., 2022; Long *et al*., 2022; Kromdijk and Walter, 2023), as lower values of NPQ were significantly correlated with faster rates of photosynthetic induction and stomatal opening (Fig. 7). Future studies could examine in greater detail if the relationship between low-light NPQ values and speed of induction still holds under many subsequent fluctuating light events.

### Future perspectives and applications of this study in high-throughput phenotyping

This study helps to lay the foundation for better understanding of whether non-steady-state CF and leaf temperature measurements can be used as a proxy for the speed of induction as quantified in changes to *A* or *g*_s_. However, future studies should assess correlations (*A*–Φ_PSII_ and *g*_s_–*T*_leaf_) across varied environmental conditions (e.g. [CO_2_], vapor pressure deficit, and air flow) and developmental stages, and further validate under which conditions CF and *T*_leaf_ can be used to make inferences about *A* and *g*_s_. One of the main applications of Φ_PSII_ and *T*_leaf_ kinetics as indicators for *A* and *g*_s_ kinetics is to screen the dynamic response across diverse and varied genotypes and select for improved photosynthetic performance under fluctuating light, using CF and thermal imaging. Unlike gas exchange, these methods can provide great spatiotemporal resolution at plant and canopy level (Baker, 2008; McAusland *et al*., 2013; Fu *et al*., 2022) and facilitate dynamic measurements. However, these methods require careful application.

One of the limitations of this study—and a future opportunity—is that leaf temperature was measured on the abaxial side of the leaf, where most stomata are typically present. Conventionally, this is thought of as the most accurate and sensitive way of measuring *T*_leaf_ as it relates directly to changes in evaporative cooling due to transpiration. However, this is difficult to deploy for larger studies in which high-throughput phenotyping methodologies would be necessary. Consequently, another connection that needs to be made is better coupling of *T*_leaf_ and boundary layer thickness on the adaxial surface—as is typically measured with infrared thermography—with the *T*_leaf_ on the abaxial surface, where more accurate measures are taken. As such, greater effort needs to be made to assess if high-throughput methods focused on the adaxial side can better predict changes occurring on the abaxial side. In addition to considerations related to differences between adaxial and abaxial leaf surfaces, parameter estimation via CF and thermal imaging is sensitive to leaf movement, leaf angle, and heterogeneous absorbance of actinic and saturating light across the field of view of the camera. Therefore, such imaging techniques best suit compact and ‘two-dimensional’ plants, such as seedlings, rosette-forming species such as Arabidopsis, and early developmental stages of crops such as lettuce, as these plants show more uniform light absorption. This has limited studies on species with diverse sizes and architectures, especially plant species with complex canopies with high incidence of mutual shading where variability for parameters such as Φ_PSII_ can increase (Baker, 2008; Flood *et al*., 2016; Lawson and Vialet-Chabrand, 2018; Bengoa Luoni *et al*., 2024).

However, recent studies have tried to tackle these variations in leaf surfaces by quantifying differences in functional anatomy, temperature, and boundary layer (Vialet-Chabrand and Lawson, 2019; Wall *et al*., 2023; J. Zhang *et al*., 2024, 2025). This has included the use of reference materials with properties similar to leaves but with several different colors (Vialet-Chabrand and Lawson, 2019, 2020) or with defined pores to resemble a range of stomatal conductance (J. Zhang *et al*., 2025). These new methods may also facilitate the capture of the kinetics of Φ_PSII_ and *T*_leaf_, as quantified here in terms of *t*_50_ or *t*_90_, with effects of local gradients on leaf surfaces accounted for through normalization of data based on the initial and final values (J. Zhang *et al*., 2024). By pairing information on the relationships between physiological parameters under fluctuating light with new methods to apply thermal imaging, we should be able to accelerate the phenotyping of photosynthetic traits in diverse germplasm in the future.

## Conclusion

The rates of change in Φ_PSII_ and *T*_leaf_ can be used as indicators of *A* and *g*_s_ kinetics, respectively. Specifically, the time to reach 50% of steady-state *A*, and the times to reach 90% of full stomatal opening as well as closure, could be screened by using CF and thermal imaging, respectively. These indicators may help overcome phenotyping bottlenecks and facilitate rapid, large-scale screening of genotypes to identify the underlying mechanisms and phenotype–genotype associations of these kinetics. This approach supports the research community in rapid evaluation of photosynthetic traits, especially faster kinetics in *A* and *g*_s_, which can potentially improve light and water use efficiency. Ultimately, this may help generate further insights as well as gene discovery to enhance crop productivity under fluctuating light conditions.

## Supplementary Material

erag231_Supplementary_Data

## Data Availability

The primary data and associated metadata are publicly available through the WUR data repository at https://doi.org/10.17887/WUR01-TMWYJN.

## Supplementary data

The following supplementary data are available at *JXB* online.

Fig. S1. Raw data of time courses of stomatal conductance and leaf temperature in Arabidopsis, showing artifactual spikes caused by saturating pulses and changes in light intensity.

Fig. S2. Example time courses and illustration of LOESS curve fitting to experimental data and determined initial and final values of measured traits.

Fig. S3. Differences (Δ) between initial and final values, measured in nine species under stepwise changes in light intensity.

Fig. S4. Values of non-photochemical quenching in nine species under stepwise changes in light intensity.

Fig. S5. Maximum quantum efficiency of PSII.

Fig. S6. Response times of non-photochemical quenching of nine species under stepwise changes in light intensity.

Fig. S7. Times to reach 63.2% (τ) of photosynthesis and stomatal conductance correlate with those of chlorophyll fluorescence and leaf temperature, respectively.

Fig. S8. Correlations between the times required for a change in net photosynthesis rate versus PSII operating efficiency per species.

Fig. S9. Correlations between the times required for a change in stomatal conductance versus leaf temperature per species when light intensity was changed from 50 to 1000 μmol m^−2^ s^−1^.

Fig. S10. Correlations between the times required for a change in stomatal conductance versus leaf temperature per species when light intensity was changed from 1000 to 50 μmol m^−2^ s^−1^.
