# Supplementary material for: Using ΦPSII and leaf temperature as indicators of non-steady-state photosynthesis and stomatal conductance during stepwise changes in light intensity
Source: J Exp Bot. 2026 May 16;77(16):5168–85. doi: 10.1093/jxb/erag231 (PMC13529326; doi:10.1093/jxb/erag231)
Supplement: Supplementary file 1 [file erag231_supplementary_data.pdf]

Using Φ_{PSII} and leaf temperature as indicators of non-steady-state photosynthesis and stomatal conductance during stepwise changes in light intensity

Elahe Javadi Asayesh[1], Leo F.M. Marcelis[1], Liana G. Acevedo-Siaca[1*], Elias Kaiser[1,2*]

[1] Horticulture and Product Physiology, Department of Plant Sciences, Wageningen University & Research, PB 6708, Wageningen, Netherlands

[2] Research Institute of Agriculture and Life Sciences, Seoul National University, Seoul, Republic of Korea

*Corresponding authors: liana.acevedosiaca@wur.nl; elias.kaiser@wur.nl

Supplementary material

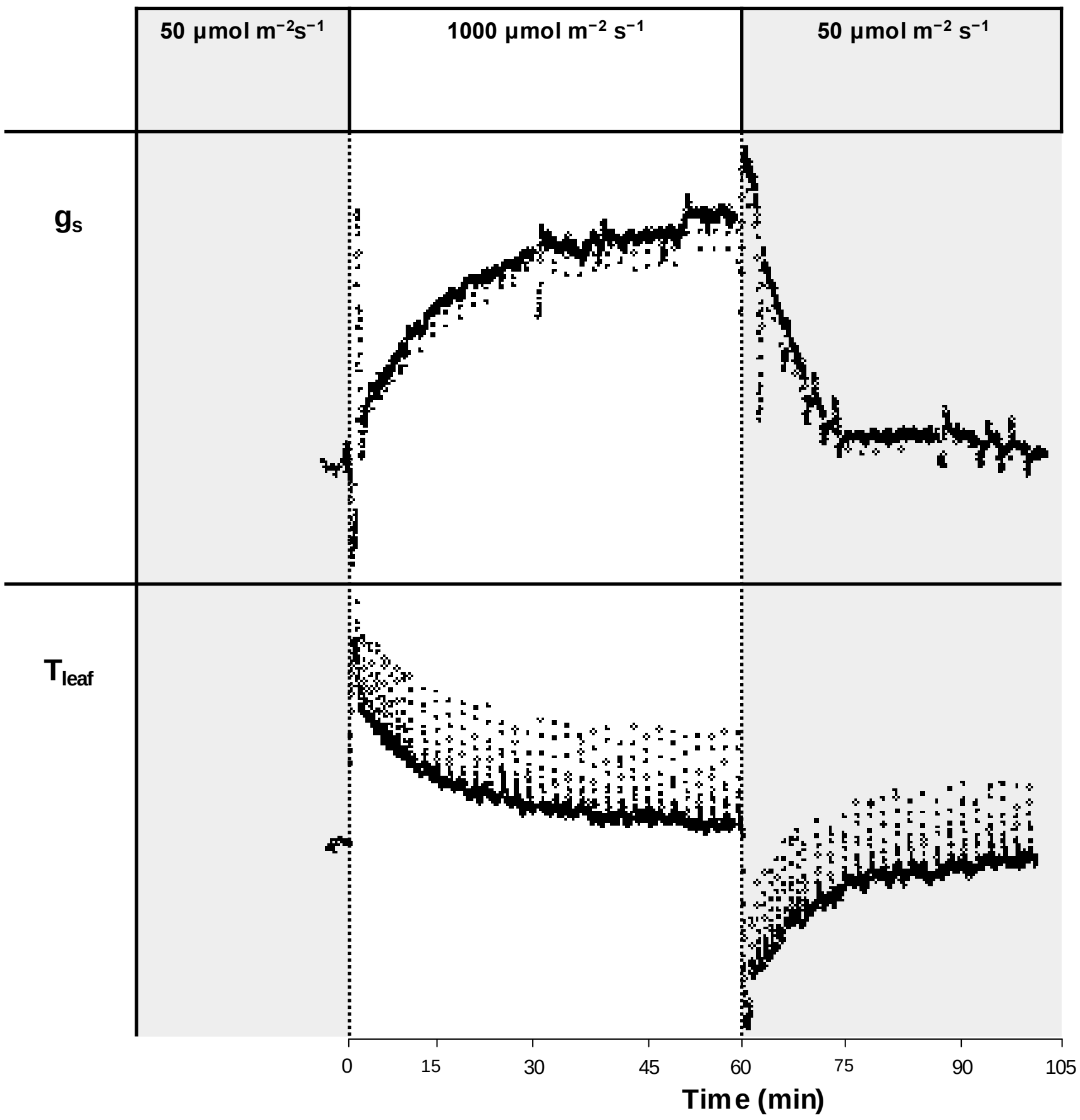

Supplementary Figure S1- Raw data of time courses of stomatal conductance (g_s) and leaf temperature (T_{leaf}) in Arabidopsis, showing artefactual spikes caused by saturating pulses and changes in light intensity.

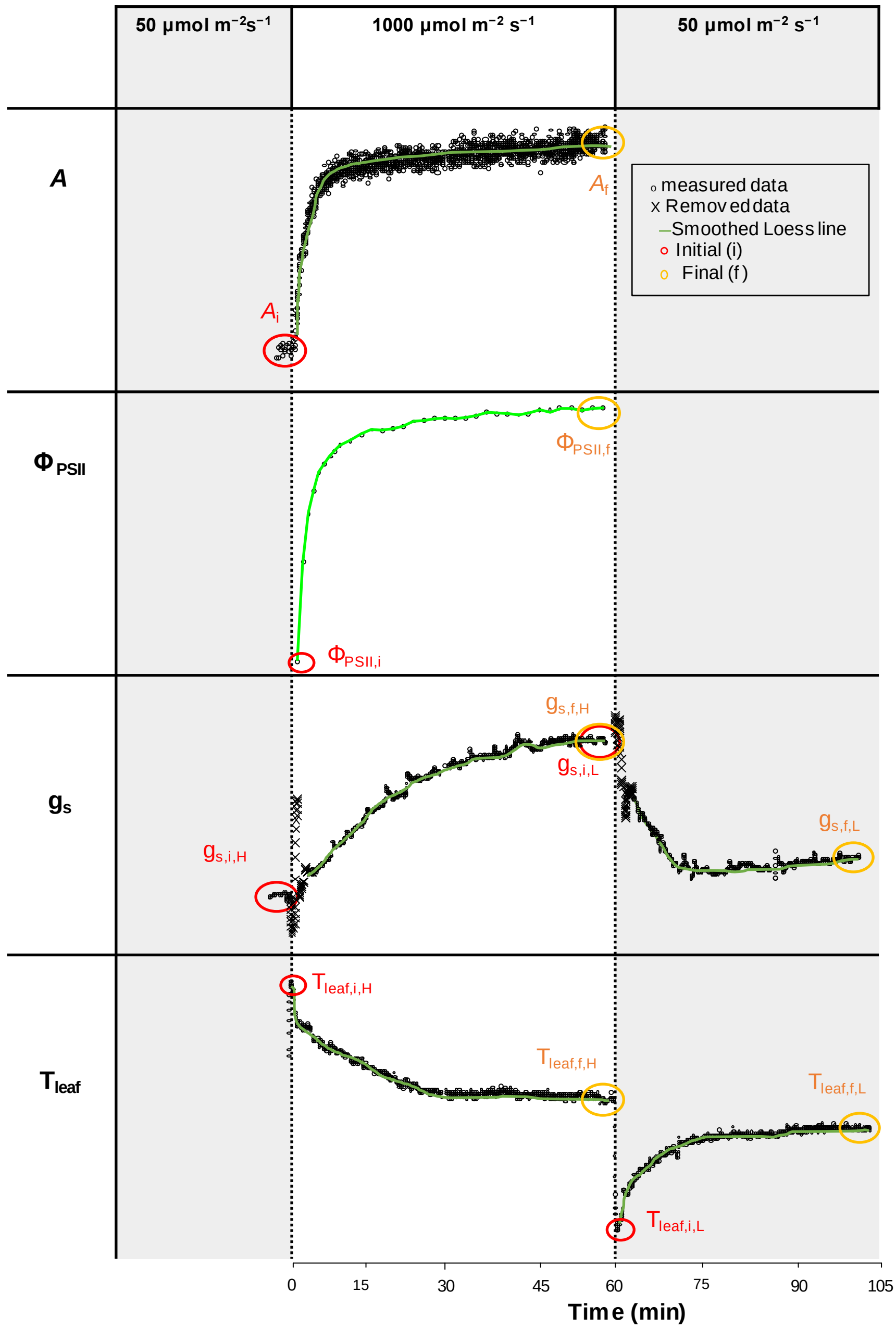

Supplementary Figure S2- Example time courses and illustration of LOESS curve fitting to experimental data and determined initial and final values of measured traits. Net photosynthesis rate (A), photosystem II operating efficiency (Φ_{PSII}), stomatal conductance (g_s), and leaf temperature (T_{leaf}) when light intensity was changed from 50 to 1000 µmol m^{-2} s^{-1} for 60 minutes (H), followed by a decrease to 50 µmol m^{-2} s^{-1} for 45 minutes L; grey shade). Dots represent measured data, crosses indicate data points removed due to spikes and rapid changes in T_{leaf}. Initial values (i; red circles) and final steady-state values (f; orange circles) are shown per trait, calculated as averages of multiple data points—except for initial Φ_{PSII} and T_{leaf}, which are based on the first data point. Green lines represent smooth curves fitted to the data using the LOESS function in R.

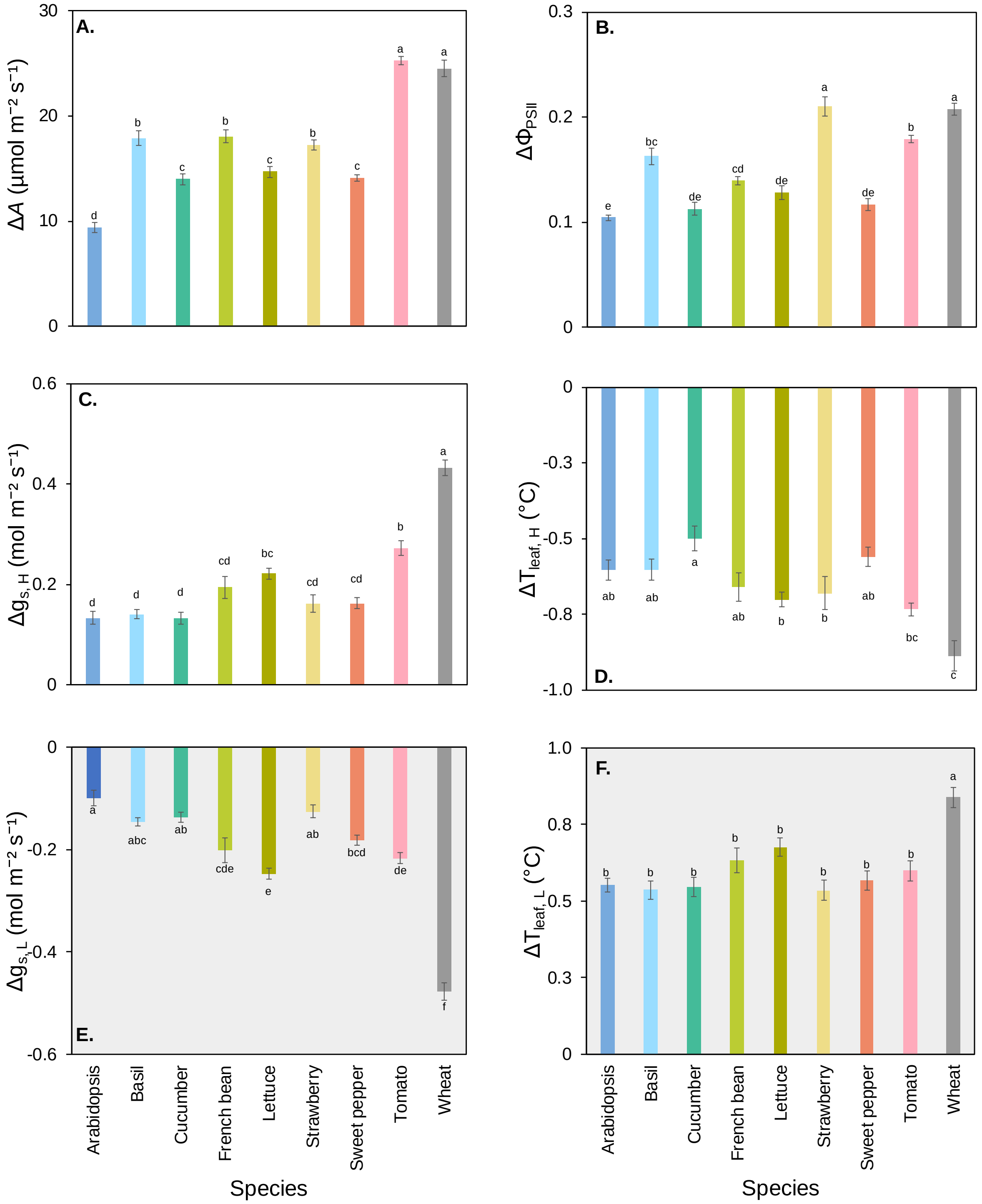

Supplementary Figure S3- Differences (Δ) between initial and final values, measured in nine species under stepwise changes in light intensity. A) net photosynthesis rate (A), B) photosystem II operating efficiency (Φ_{PSII}), C and E) stomatal conductance (g_s), D and F) leaf temperature (T_{leaf}). Panels A-D show values related to the 50 to 1000 µmol m^{-2} s^{-1} shift (H), panels E-F (grey background) show values related to the 1000 to 50 µmol m^{-2} s^{-1} shift (L). Bars display means ± SE (n = 9), letters represent significant differences between species ($p<0.05$).

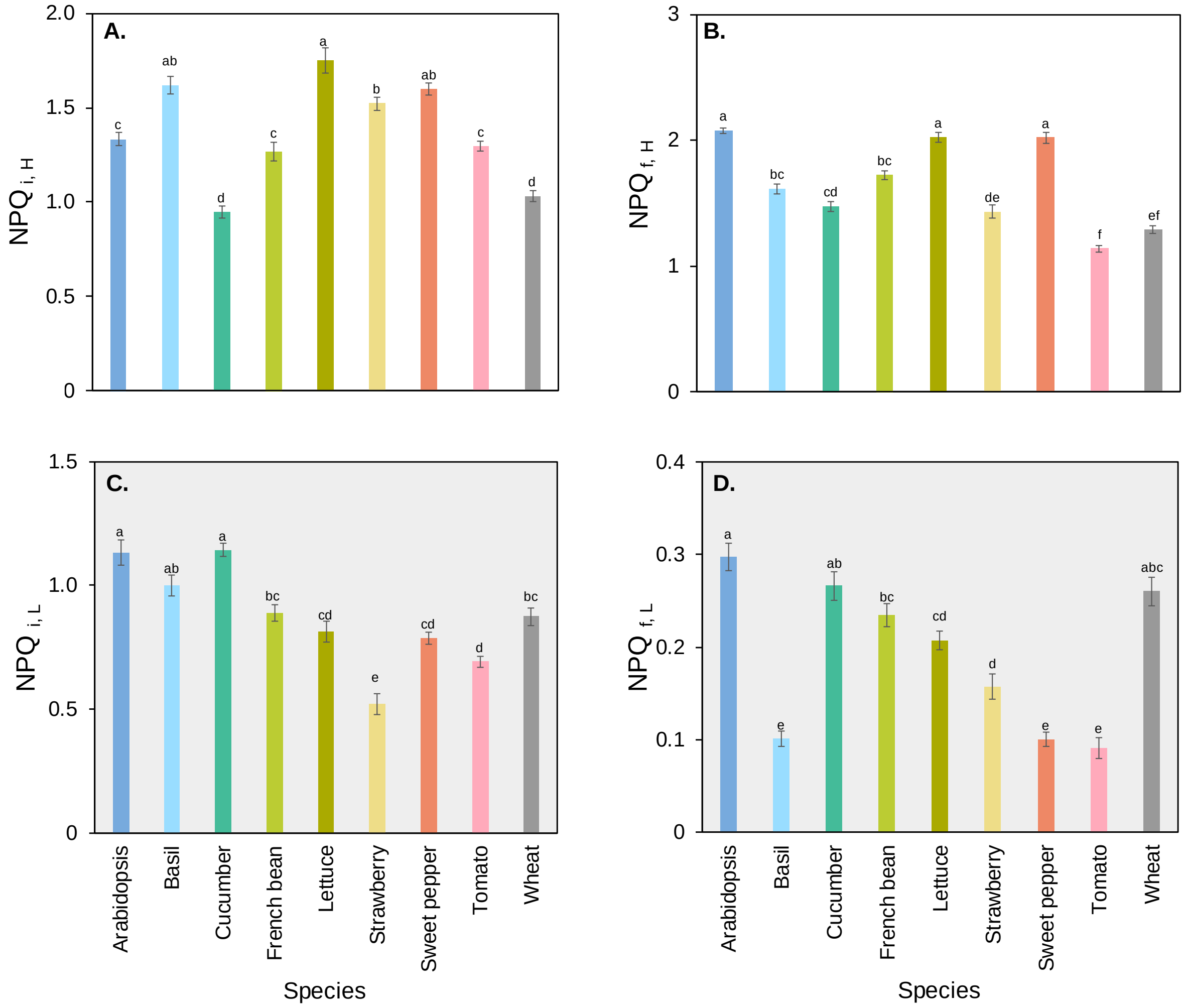

Supplementary Figure S4- Values of non-photochemical quenching (NPQ) in nine species under stepwise changes in light intensity. A) Initial NPQ value as the first data point (NPQ_i) and B) final NPQ value calculated from the average of the last two data points (NPQ_f) under light intensity of 1000 µmol m^{-2} s^{-1} (H) and C) NPQ_i and D) NPQ_f under light intensity of 50 µmol m^{-2} s^{-1} (L, grey background). Bars display means ± SE (n = 9), letters represent significant differences between species ($p<0.01$).

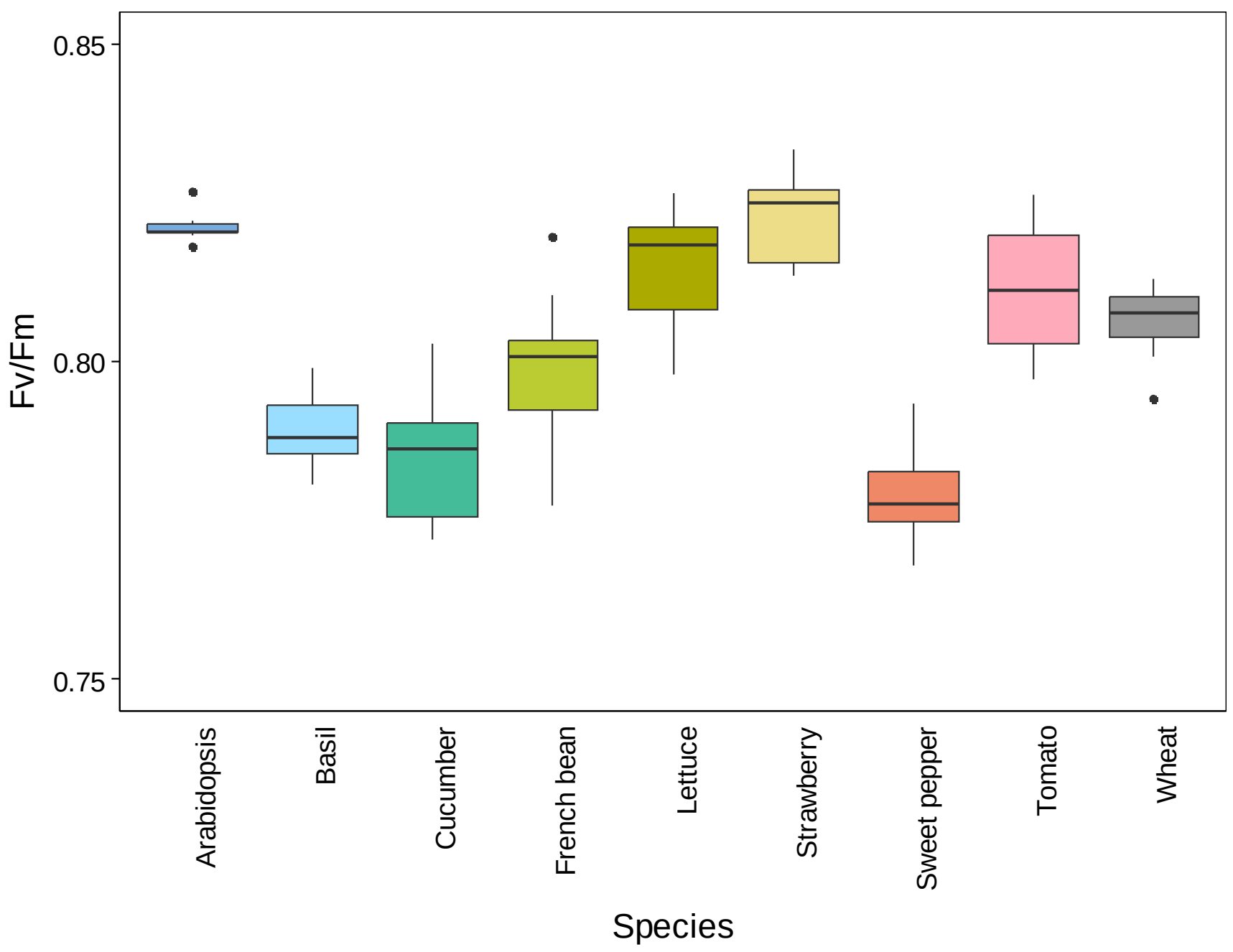

Supplementary Figure S5- Maximum quantum efficiency of PSII (F_v/F_m). Horizontal line inside each box displays the median of nine replicates (n=9) per species.

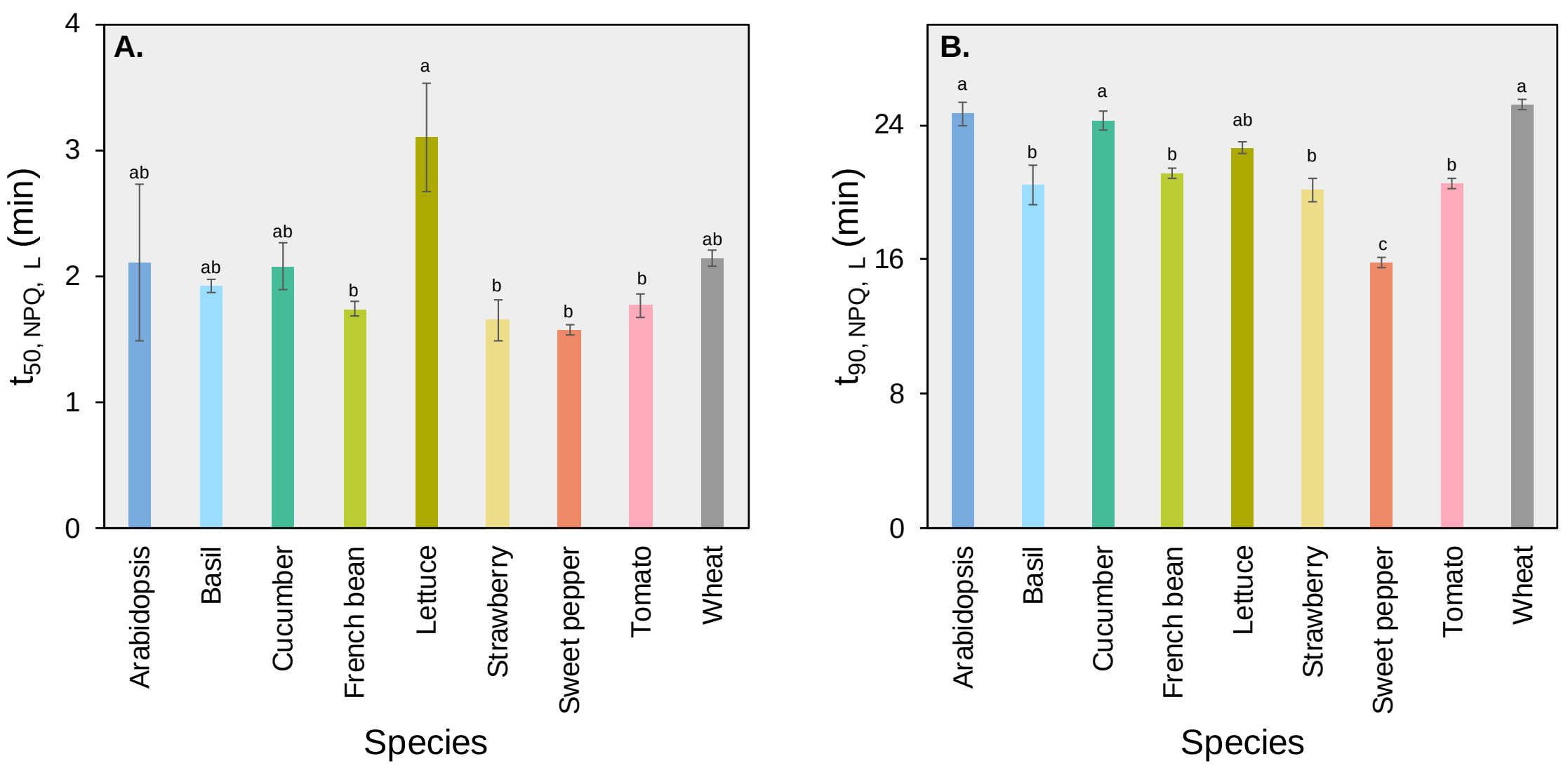

Supplementary Figure S6- Response times of non-photochemical quenching (NPQ) of nine species under stepwise changes in light intensity. A) time to reach 50% (t_{50}) and B) time to reach 90% (t_{90}) of the final steady-state NPQ when light intensity was changed from 1000 to 50 µmol m^{-2} s^{-1} (L, grey background). Bars display means ± SE (n = 9), letters represent significant differences ($p<0.001$).

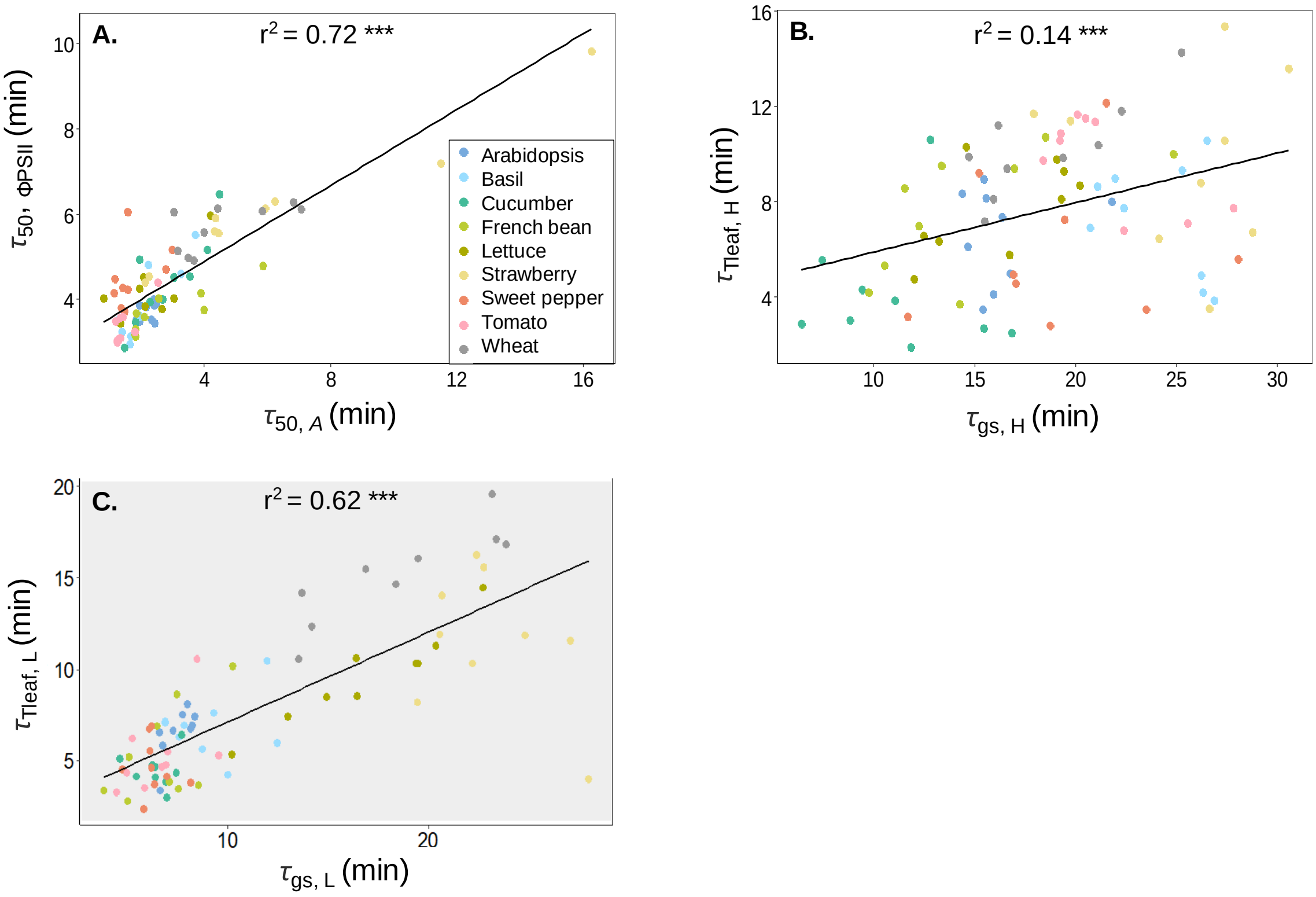

*Supplementary Figure S7- Times required for change of photosynthesis and stomatal conductance correlate with those of chlorophyll fluorescence and leaf temperature, respectively. Panels show correlations between times to reach 63.2% (τ) of the final value per given trait. A) time for net photosynthesis rate (A) versus photosystem II operating efficiency (Φ_{PSII}) and B) stomatal conductance (g_s) versus leaf temperature (T_{leaf}) when light intensity was changed from 50 to 1000 µmol m^{-2} s^{-1} (H), and C) g_s versus T_{leaf} when transitioning from 1000 to 50 µmol m^{-2} s^{-1} (L; grey background). Each dot represents a biological replicate, with nine replicates per nine species (n = 81). Associated P-value and r^2 are shown per correlation. Asterisks indicate statistical significance levels: * $p < 0.05$; ** $p < 0.01$; *** $p < 0.001$.*

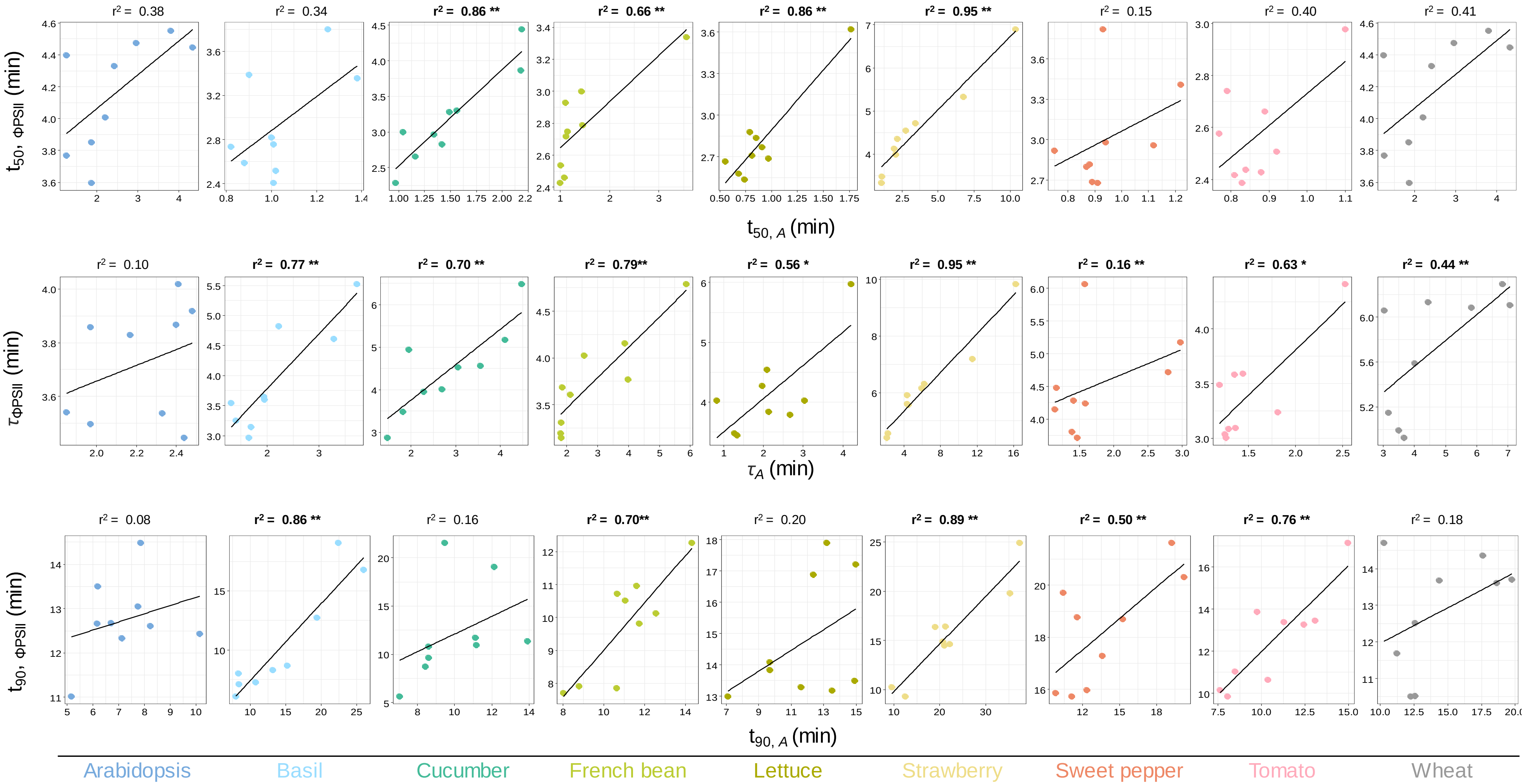

*Supplementary Figure S8- Correlations between the times required for a change in net photosynthesis rate (A) versus photosystem II operating efficiency (Φ_{PSII}) per species. Panels show correlations between times to reach 50% (t_{50}; top), 63.2% (τ, middle) and 90% (t_{90}; bottom) of the final value for A versus Φ_{PSII} when light intensity was changed from 50 to 1000 µmol m^{-2} s^{-1}. Each dot represents a biological replicate, with nine replicates per nine species (n=9). Associated P-value and r^2 are shown per correlation with significant correlations in bold. Asterisks indicate statistical significance levels: * $p < 0.05$; ** $p < 0.01$; *** $p < 0.001$.*

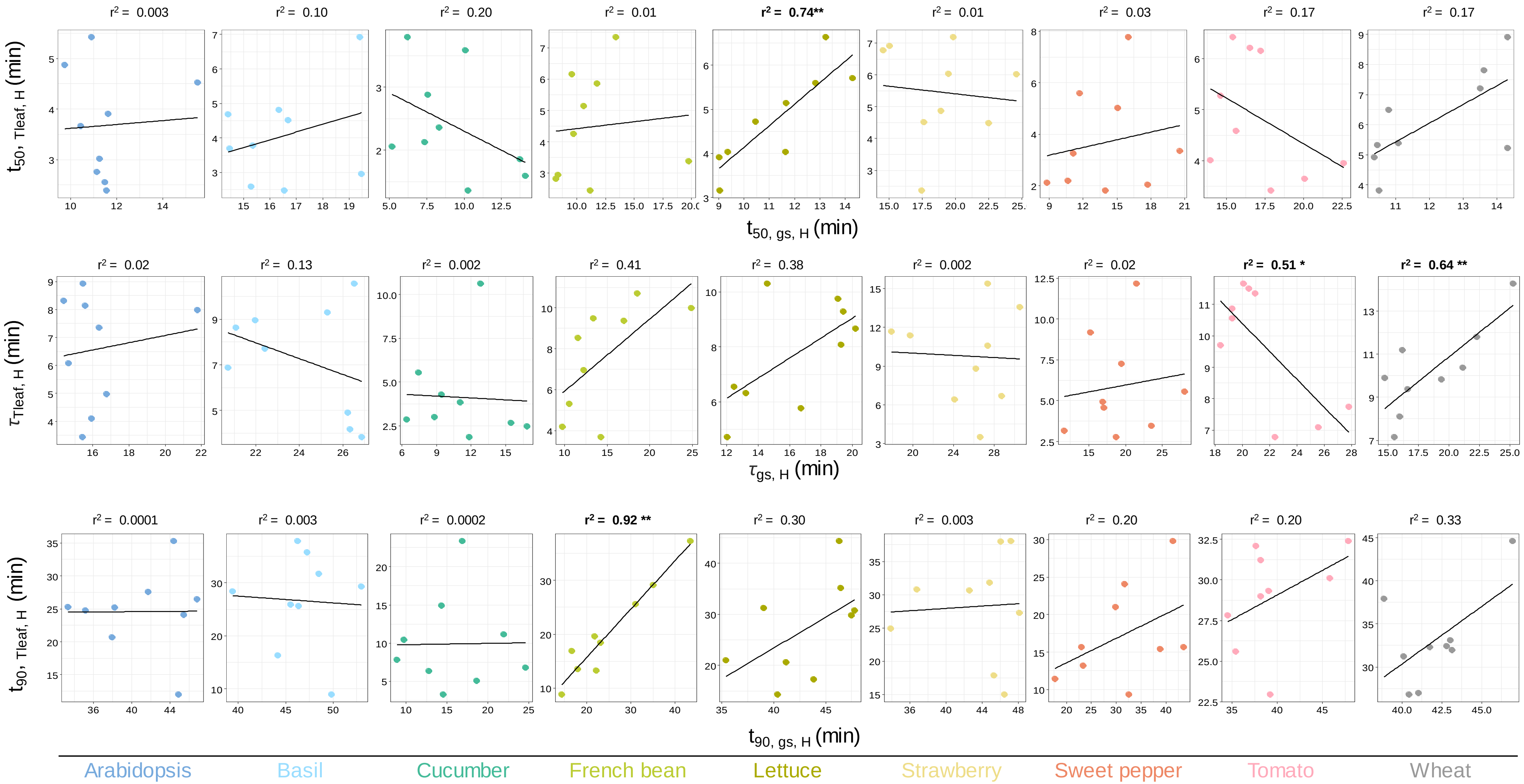

*Supplementary Figure S9- Correlations between the times required to change in stomatal conductance (g_s) versus leaf temperature (T_{leaf}) per species. Panels show correlations between times to reach 50% (t_{50}; top), 63.2% (τ, middle) and 90% (t_{90}; bottom) of the final value for g_s versus T_{leaf} when light intensity was changed from 50 to 1000 $\mu mol\ m^{-2}\ s^{-1}$ (H). Each dot represents a biological replicate, with nine replicates per nine species (n=9). Associated P-value and r^2 are shown per correlation with significant correlations in bold. Asterisks indicate statistical significance levels: * $p < 0.05$; ** $p < 0.01$; *** $p < 0.001$.*

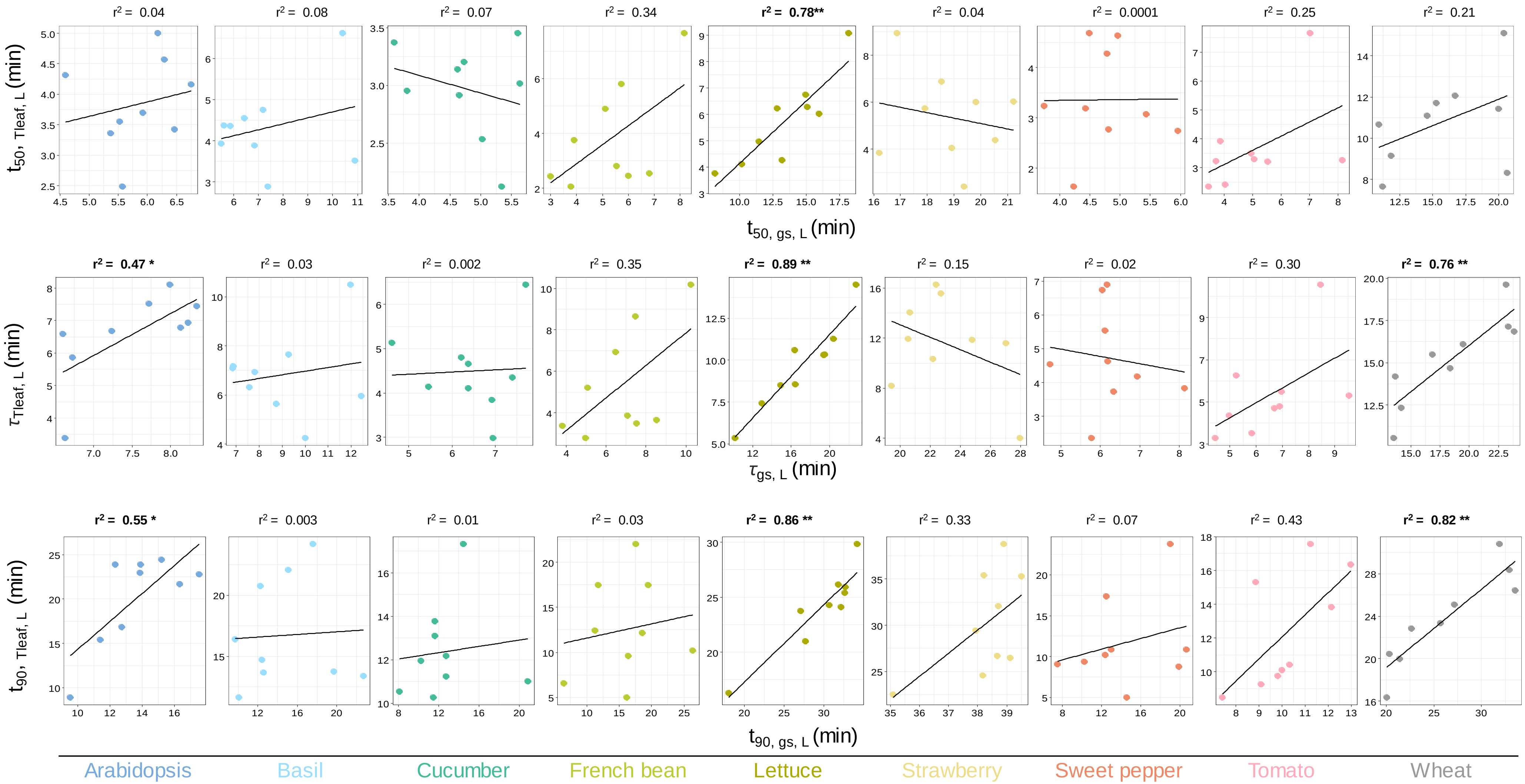

*Supplementary Figure S10- Correlations between the times required to change in stomatal conductance (g_s) versus leaf temperature (T_{leaf}) per species. Panels show correlations between times to reach 50% (t_{50}; top), 63.2% (τ, middle) and 90% (t_{90}; bottom) of the final value for g_s versus T_{leaf} when light intensity was changed from 1000 to 50 µmol m^{-2} s^{-1} (L). Each dot represents a biological replicate, with nine replicates per nine species (n=9). Associated P-value and r^2 are shown per correlation with significant correlations in bold. Asterisks indicate statistical significance levels: * $p < 0.05$; ** $p < 0.01$; *** $p < 0.001$.*
